# Impact of Electronic Cigarette Use on the Oral Microbiota: A Systematic Review

**DOI:** 10.1111/jcpe.70111

**Published:** 2026-03-19

**Authors:** Giusy Rita Maria La Rosa, Lakshman Perera Samaranayake, Egija Zaura, Ang Sun, Virginia Fuochi, Pio Maria Furneri, Jan Kowalski, Karol Myszel, Iain Chapple, Riccardo Polosa

**Affiliations:** ^1^ Department of Clinical and Experimental Medicine University of Catania Catania Italy; ^2^ The University of Hong Kong Pokfulam Hong Kong; ^3^ Center of Excellence in Periodontal Disease and Dental Implantology, Faculty of Dentistry Chulalongkorn University Bangkok Thailand; ^4^ Global Research Cell, Dr. D. Y. Patil Dental College and Hospital, Dr. D. Y. Patil Vidyapeeth, Pimpri Pune India; ^5^ Department of Preventive Dentistry, Academic Centre for Dentistry Amsterdam University of Amsterdam and Vrije Universiteit Amsterdam Amsterdam the Netherlands; ^6^ Department of Biology, College of Science and Technology Temple University Philadelphia Pennsylvania USA; ^7^ Sbarro Institute for Cancer Research and Molecular Medicine, Center for Biotechnology, College of Science and Technology Temple University Philadelphia Pennsylvania USA; ^8^ Department of Biomedical and Biotechnological Sciences (BIOMETEC) University of Catania Catania Italy; ^9^ Department of Periodontology Medical University of Warsaw Warsaw Poland; ^10^ Center of Hearing and Speech Kajetany Poland; ^11^ Faculty of Health Sciences University of Applied Sciences Konin Poland; ^12^ Periodontal Research Group & Birmingham NIHR Biomedical Research Centre in Inflammation The University of Birmingham & Birmingham Community Healthcare NHS Trust Birmingham UK; ^13^ Center for the Acceleration of Harm Reduction University of Catania Catania Italy

**Keywords:** dysbiosis, e‐cigarette, oral microbiota, periodontal disease, smoking

## Abstract

**Aim:**

To examine whether the oral microbiota of e‐cigarette users differs from that of never smokers and current smokers.

**Materials and Methods:**

PubMed, Scopus and Web of Science were searched on 27 August 2025. Human studies using molecular methods to compare oral microbiota in saliva, subgingival plaque and oral mucosal swabs among e‐cigarette users, never smokers and current smokers were included. Primary outcomes were alpha diversity, beta diversity and differential taxonomic abundance. Risk of bias was assessed using JBI tools and certainty of evidence using GRADE.

**Results:**

Twelve studies were included; most were cross‐sectional and heterogeneous in design, exposure and samples. Alpha diversity findings were inconsistent across samples, whereas beta diversity more consistently indicated distinct microbial communities in e‐cigarette users compared with never smokers and current smokers. Taxonomic differences were heterogeneous and sample‐dependent, with some enrichment of genera such as *Veillonella*, *Leptotrichia*, and *Fusobacterium* compared with never smokers.

**Conclusions:**

Electronic cigarette use is associated with sample‐specific oral microbiota differences that partly overlap with, but differ from, those observed in never smokers and current smokers. However, the certainty of evidence is very low due to predominantly cross‐sectional designs and methodological limitations, underscoring the need for longitudinal studies with standardised exposure and protocols.

## 1 Introduction

Electronic cigarettes are increasingly used as smoking cessation tools, supported by evidence of their effectiveness in helping individuals quit combustible cigarettes (Lindson et al. 2025; O'Leary et al. 2025). While the harmful effects of conventional smoking are well known and mainly linked to combustion products (Varghese and Muntode Gharde 2023), the long‐term consequences of e‐cigarette aerosol exposure and therefore safety—especially within the oral cavity—remain incompletely understood.

The oral cavity, as the first point of contact with inhaled substances, harbours a diverse microbiota of over 700 bacterial species alongside fungal and viral communities (Kilian et al. 2016; Zhang et al. 2018; Caselli et al. 2020). This ecosystem contributes to oral and systemic health through immune regulation and homeostasis (Deo and Deshmukh 2019; Shigdel et al. 2023; Costa et al. 2024) and is highly sensitive to external influences such as diet, medication, oral hygiene and tobacco exposure (Marsh 1994; Benn et al. 2022; Chattopadhyay et al. 2024; Fan et al. 2024; Nath et al. 2025). Dysregulation of this system has been linked to oral and systemic diseases, including periodontitis and atherosclerosis (Wang et al. 2019; Kroese et al. 2021; Akhi et al. 2025; La Rosa, Lorenzo‐Pouso, et al. 2025).

While the impact of conventional smoking on the oral microbiota is well established—favouring anaerobic and facultative anaerobic pathogens and associated inflammation (Mason et al. 2015; Jia et al. 2021)—the effects of e‐cigarette use are less clear. Some studies report milder alterations (Stewart et al. 2018; Thomas et al. 2022; Liu et al. 2025), whereas others show increased levels of periodontitis‐associated taxa and exacerbated inflammatory responses (Pushalkar et al. 2020; Xu et al. 2022; Miluna‐Meldere et al. 2024). Such inconsistencies may derive from variations in sequencing methodology and confounding due to the inclusion of dual or former smokers.

This is the first systematic review to critically evaluate the clinical evidence on how e‐cigarette use affects the oral microbiota, comparing e‐cigarette users, current smokers and never smokers, and identifying methodological gaps to guide future research.

## 2 Materials and Methods

The protocol of this systematic review was registered in the PROSPERO database (CRD420251120281) and previously published (La Rosa, Samaranayake, et al. 2025). The present review was reported in accordance with the Preferred Reporting Items for Systematic Review and Meta‐Analysis Protocols (PRISMA 2020) guidelines for systematic reviews and meta‐analyses (Page et al. 2021).

### 2.1 Research Question

This systematic review addressed the following research question: ‘Does the oral microbial profile of e‐cigarette users differ from that of never smokers and current smokers?’

The review was structured according to the PECO framework:

- Population: Human adults (aged 18 years and older)
- Exposure: Use of electronic cigarettes, regardless of device type, frequency or duration
- Comparison: Never smokers and/or current smokers
- Outcome: Measures of microbial diversity (e.g., alpha and beta diversity) and relative abundance of microbial taxa.

### 2.2 Search Strategy

A comprehensive literature search was conducted to identify all relevant studies published between January 2010—the year when the first clinical trials on e‐cigarettes became available—and 27 August 2025. Searches were performed across three electronic databases: PubMed, Scopus and Web of Science (La Rosa, Samaranayake, et al. 2025). The full search strategies are provided in Appendix 1.

Secondary search strategies, including reference list screening, citation tracking and targeted grey literature searching (i.e., OpenGrey database), were employed to minimise publication bias. Detailed procedures are described in the published protocol (La Rosa, Samaranayake, et al. 2025). No language or publication date restrictions were applied.

### 2.3 Study Selection

Eligibility criteria were defined according to study design, population, exposure and methodology, as detailed in the published protocol (La Rosa, Samaranayake, et al. 2025). We included randomized controlled trials (RCTs) and observational studies assessing the oral microbiota in adult e‐cigarette users (≥ 18 years), with never smokers or current smokers as comparators, using molecular microbial methods. Studies based on culture techniques, animal or in vitro models, lacking comparator groups, or not reporting primary data were excluded. Dual use (i.e., concomitant e‐cigarette and cigarette use) was considered a variation of e‐cigarette exposure and, when clearly reported, was analysed as a separate subgroup within e‐cigarette users.

Title and abstract screening were independently conducted by two reviewers (G.R.M.L.R., R.P.) using EndNote 21, with full‐text assessment based on predefined eligibility criteria. Discrepancies were resolved through discussion or by consulting a third reviewer (L.P.S.). Inter‐reviewer agreement during study selection was high (Cohen's κ = 0.93).

### 2.4 Data Extraction

Data extraction was independently performed by two reviewers (G.R.M.L.R., J.K.) using a standardised form developed and piloted according to the published protocol (La Rosa, Samaranayake, et al. 2025). Extracted information included study design, setting, sample characteristics, exposure details and microbial analysis method (e.g., sample type and collection method, DNA extraction method, taxonomy databases and software employed for bioinformatics analysis). Primary outcomes were microbial alpha and beta diversity and differential relative abundance of taxa. For quantitative polymerase chain reaction (qPCR)‐based studies, microbial changes were reported exactly as presented in the original articles, for example, 16S rRNA gene copy numbers or other quantitative measures of selected taxa. Any discrepancies were resolved by discussion or consultation with a third reviewer (I.C.).

### 2.5 Bias Assessment

The risk of bias was independently assessed by two reviewers (G.R.M.L.R. and R.P.) using the Joanna Briggs Institute (JBI) Critical Appraisal Tools, selecting the appropriate checklist according to the design of each study (https://jbi.global/critical‐appraisal‐tools). Further details on the microbiota‐specific criteria applied are reported in Appendix 2. Disagreements between reviewers were resolved by consensus or, when necessary, through consultation with a third reviewer (E.Z.).

### 2.6 Data Synthesis

Findings were narratively synthesised and summarised in tables to facilitate comparison of microbial diversity and taxonomic composition across e‐cigarette users, never smokers and current smokers. Where possible, the direction and magnitude of observed differences were reported alongside key methodological features influencing results.

Raw‐data harmonisation was not performed because it was not appropriate for the aims of this review. No quantitative meta‐analysis was planned because of the predominance of cross‐sectional designs and substantial clinical and methodological heterogeneity across studies. In addition, raw sequencing data were not consistently available, and harmonising only a subset would have introduced selective bias. Accordingly, the review was based on a critical narrative synthesis of results as reported in the original publications.

#### 2.6.1 Subgroup Analyses

Pre‐specified subgroup analyses (La Rosa, Samaranayake, et al. 2025) explored potential moderators of the association between e‐cigarette use and oral microbiota alterations, including oral disease status, user type (exclusive vs. dual) and sample type (saliva, subgingival plaque, mucosa).

### 2.7 Certainty of the Evidence

The Grading of Recommendations, Assessment, Development, and Evaluation (GRADE) approach was applied by two independent reviewers (G.R.M.L.R. and R.P.) to assess the certainty of evidence for the three main outcomes (alpha diversity, beta diversity and differential taxa abundance). As no meta‐analysis was conducted, GRADE was applied following the guidance for narrative synthesis proposed by Murad et al. (2017).

## 3 Results

### 3.1 Search Results

**FIGURE 1:**
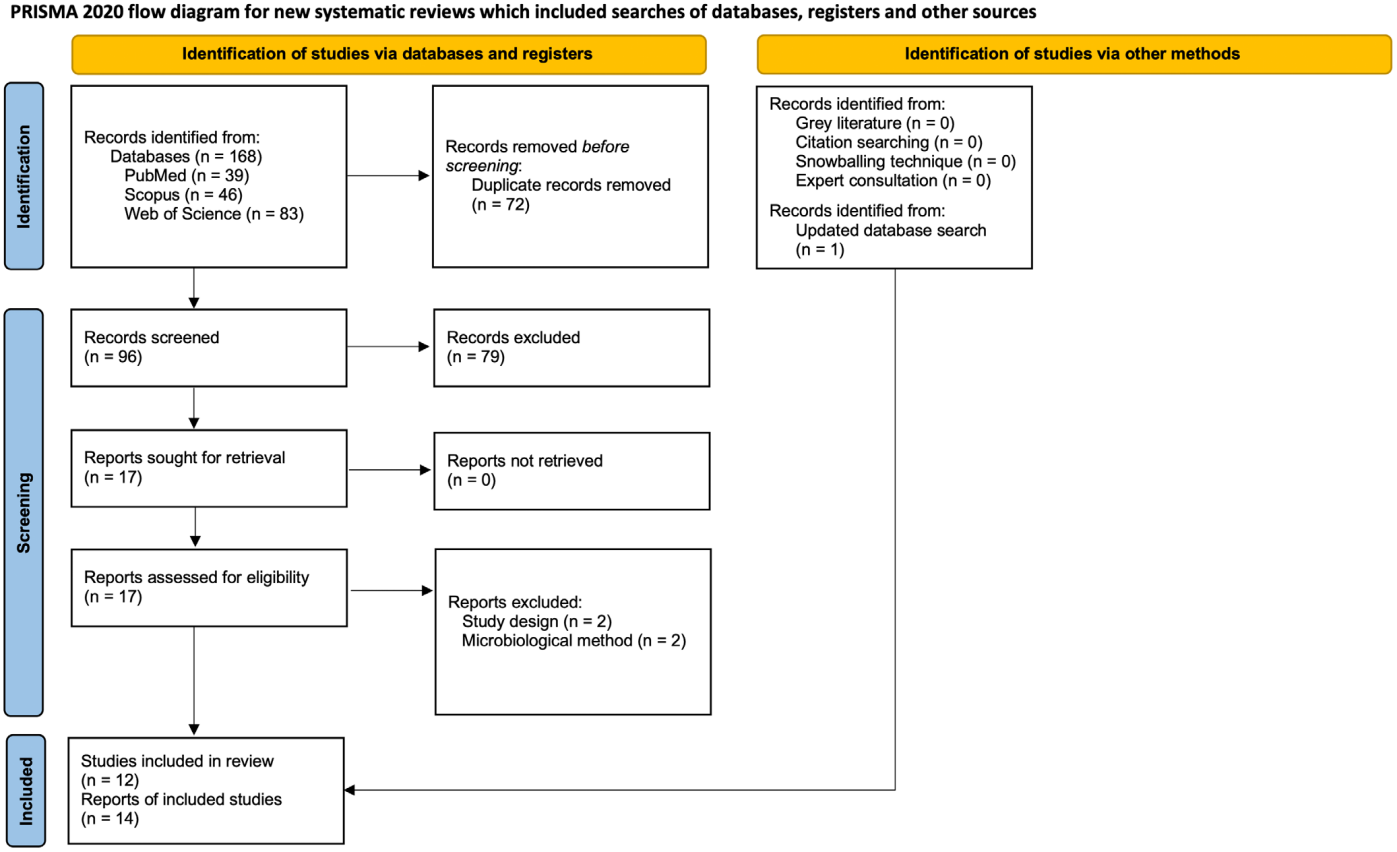
PRISMA 2020 flow diagram of the search process.

The study selection process is summarised in the PRISMA flowchart (Figure 1). After duplicate removal, 96 records were screened by title and abstract, yielding 17 full‐text articles for eligibility assessment. Of these, four studies were excluded (Adam and Hasan 2023; Coll and Geddes 2023; Aldakheel et al. 2020; Cichonska et al. 2021). The list of the excluded studies after full‐text screening and the reasons for exclusion are reported in Appendix 3. A total of 11 studies, reported across 13 publications, met the inclusion criteria and were included in the qualitative synthesis (Stewart et al. 2018; Thomas et al. 2022; Liu et al. 2025; Pushalkar et al. 2020; Xu et al. 2022; Miluna‐Meldere et al. 2024; Chopyk et al. 2021; Ganesan et al. 2020; Kurniawan et al. 2025; Park et al. 2023; Wang et al. 2022; Yang et al. 2023; Ying et al. 2022). An updated literature search conducted on 31 January 2026 identified one additional eligible study (Yang et al. 2025). Overall, 12 studies, reported in 14 publications, were ultimately included in the review (Table 1). Three publications (Thomas et al. 2022; Pushalkar et al. 2020; Xu et al. 2022) originated from the same cohort and were therefore considered a single study.

**TABLE 1:** General characteristics of the included studies.

Author (year), country	Study design	Sample size	Participant characteristics (mean age ± SD/female %)	E‐cigarette status	Control group(s) status	Smoking status verification	Oral health status	Subject preparation	Outcomes	Funding
Stewart et al. (2018), USA	Cross‐sectional	*N* = 30 (NS = 10; CS = 10; EC = 10)	NS, median (IQR): 31 (28–36)/10% F CS: 35 (30–45)/0% F EC: 29 (24–37)/10% F Most of participants were meat eaters (NS: 90%, CS: 100%, EC: 90%) and White (NS: 60%, CS: 60%, EC: 70%)	EC: Daily use of e‐cig for at least 6 months DU: 1% Cig/day, median (IQR): 0.2 (0.2–0.2) E‐cig use: Volume (mL)/day = 8 (3–19) Years using = 3 (2–4) Nicotine concentration (mg) = 9 (6–12)	NS	Exhaled CO breath test	NR	NR	Alpha diversity (observed OTUs, Shannon); Beta diversity (Weighted UniFrac); taxonomic differential abundance analysis (phylum and genus)	NCI, the Veteran Health Administration and the McNair Medical Institute
Ganesan et al. (2020), USA	Cross‐sectional	*N* = 123 (NS = 25; CS = 25; EC = 20; DU = 28; FS = 25)	NS: 26.2 ± 6.7/76% F CS: 22.4 ± 5.4/52% F EC: 23.2 ± 3.9/50% F DU: 26.3 ± 5.9; 60.7% F FS: 25.2 ± 7.4; 52% F ~90% Caucasian *Exclusion criteria*: Diabetes, HIV, immune‐suppressants/bisphosphonates/steroids, recent antibiotics or dental cleaning (< 3 months), < 20 teeth	EC: Daily e‐cig use ≥ 3 months, ≥ 1 cartridge or 1 mL liquid/day EC: Nicotine 6–18 mg for 4–12 months (mean ± SD, 1.1 ± 0.6 years) DU: 4–30 months FS switched EC: 6–24 months (1.2 ± 0.7 years) and quitting between 3 months and 1 year before study	NS: < 100 cigs lifetime, none past year CS: ≥ 5 pack‐years, no EC use Cig use (mean ± SD, years: 6.1 ± 2.7; package/day: 1.2 ± 0.6)	Self‐reported	Healthy periodontally (attachment loss ≤ 1; < 3 sites probing depth 4 mm; bleeding index ≤ 20%)	NR	Alpha diversity (Shannon, Chao1, ACE); beta diversity (Bray–Curtis, Jaccard); taxonomic differential abundance analysis (phylum, genus and species)	NIH and U.S. Food and Drug Administration
Pushalkar et al. (2020) Thomas et al. (2022) Xu et al. (2022), USA	Cross‐sectional and 6‐month longitudi‐nal cohort	Baseline *N* = 119 (NS = 39; CS = 40; EC = 40) Fw (6‐month)^a^: *N* = 84 (NS = 29; CS = 27; EC = 28)	Baseline NS: 33.6 ± 9.95/43.6%; CS: 45.5 ± 11.0/20%; EC: 35.9 ± 13.1/22.5% Fw: NS: 33.8 ± 11.0/44.83%; CS: 49.6 ± 9.9/18.52%; EC: 37.8 ± 10.8/21.43% Most of people were Asian (43.6%, NS), Black (50%, CS) and White (60%, EC). Mean (SD) drinks/day was similar among the three cohorts [3.0 (2.8)] *Exclusion criteria*: Uncontrolled systemic diseases, recent antibiotic use or dental cleaning (< 1 month), pregnancy/lactation, history of head/neck radiation	EC: 0.5–1 e‐cig/day ≥ 6 months; never cigarette use E‐cig/day [median/(IQR)]: 0.5 (1.0)	NS: Never smoked /used e‐cig (CO level < 7 parts ppm) CS: ≥ 10 cig/day ≥ 12 months; no e‐cig use Cig/day [median/(IQR)]: 11.0 (5.0)	Exhaled CO breath test and salivary cotinine levels	Periodontal patients^b^ Baseline: MNS 5.1%, MoNS 66.7%, SNS 28.2%; MCS 0%, MoCS 27.5%, SCS 72.5%; MEC 7.5%, MoEC 50.0%, SEC 42.5% Fw: MNS 5%, MoNS 50%, SNS 45%; MEC 0%, MoEC 45%, SEC 55%; MCS 0%, MoCS 18%, SCS 82% People with oral mucosal lesions and < 18 teeth (< 8 posterior) were excluded	NR	Alpha diversity (observed OTUs, Chao1, Shannon, Faith's PD); Beta diversity (Bray–Curtis); taxonomic differential abundance analysis (phylum, genus and species)	NIH grants and NYU Mega grant initiative
Chopyk et al. (2021), USA	Cross‐sectional	*N* = 24 (NS = 12; EC = 12)	NS: 21/75% F; EC: 21/0% F. Most people was Asian (non‐Hispanic) in both groups (50% EC, 58% NS) *Exclusion criteria*: Use marijuana more than once a month, or use any illicit drugs	EC: ≥ 0.5–1 mL e‐liquid/day (3.5–7 mL/week) ≥ 6 months; ≤ 1 cig/month in past 6 months E‐cig use: Mean duration = 1.5 years; frequency = 6.3 days/week, 26.7 times/day; mean [range] nicotine concentration = 21.3 mg/mL [5.1–37.6]	NS: Young adults; never vaped (> 1/month) or smoked (none in past year, never > 1/month prior)	Self‐reported	NR	No eating, no drinking, no brushing teeth, no chewing gum before sampling	Alpha (Observed OTUs, Faith's PD); Beta (Bray–Curtis, Jaccard); taxonomic differential abundance analysis (phylum, genus and species)	American Heart Association beginning grant‐in‐aid, NIH, ATS Foundation Award for Outstanding Early Career Investigators and Tobacco‐Related Disease Research Program grant
Wang et al. (2022), China	Cross‐sectional	*N* = 33 (NS = 6; CS = 14; EC = 5; FS = 8)	NS: 41.33 ± 12.21 CS: 37.52 ± 9.19 EC: 36.18 ± 11.62 FS: 33.09 ± 4.64 No antibiotics use in the 6 months before sampling	NR	NR	Self‐reported	People with halitosis, xerostomia, oral diseases (abscess, bleeding, periodontitis), precancerous/cancerous lesions, candidiasis, ≥ 8 missing teeth, gum bleeding > 10% sites were excluded	No eating, no drinking, no chewing gum, no smoking 30 min before sampling	Alpha diversity (Chao1, ACE, Shannon, Simpson); beta diversity (Bray–Curtis); taxonomic differential abundance analysis (phylum, genus and species)	The Research Foundation of China Tobacco Company and the Research Foundation of China Tobacco Yunnan Industrial Co. Ltd
Ying et al. (2022), USA	Cross‐sectional	*N* = 28 (NS = 10; CS = 8; EC = 10)	NS: 25.6 ± 2.8/40% F CS: 25.9 ± 2.7/12.5% F EC: 27.5 ± 1.9/40% F White were predominant among the three groups (CS = 100%, NS and EC = 80%) *Exclusion criteria*: Pulmonary/kidney/liver diseases, immune system disorders, regular marijuana use (> 10 times lifetime), marijuana use in prior 3 months, pregnancy	Daily/frequent e‐cig use ≥ 6 months; no cigarette smoking in past ≥ 6 months E‐cig use: Frequency = 147.5 (80–500) puffs/day; mean nicotine concentration = 13.4 (3–36) mg/mL 80% of EC were FS, 3.8 (0.5–10) years since last cigarette, 4.3 (0–15) years smoking, 9.1 (0–20) cig/day	NS: < 100 cigarettes lifetime; no smoking or e‐cig use in past ≥ 1 year CS: ≥ 100 cigarettes lifetime; smoking ≥ 5 cigarettes/day for ≥ 6 months; no e‐cig use within past ≥ 1 year 7.9 (0.3–13) years smoking, 18.1 (10–20) cig/day	Self‐reported	NR	NR	Alpha diversity (observed OTUs, Shannon, Gini‐Simpson); Beta diversity (Bray–Curtis); taxonomic differential abundance analysis (phylum, genus and species)	The NCI, the FDA Center for Tobacco Products, the National Center for Advancing Translational Sciences, Pelotoni Intramural Research Funds, and the Prevent Cancer Foundation This work was supported in part by the Intramural/Extramural research program of the National Center for Advancing Translational Sciences, National Institutes of Health
Park et al. (2023), USA	Cross‐sectional	*N* = 150 (NV = 75; EC = 75).	NS, median (IQR): 25 (21–28)/27% F EC: 23 (20–27)/19% F *Exclusion criteria*: Any tobacco use in the last 3 months and more of 5 pack‐years exposure to tobacco use; any dental cleaning in the last 3 months, antibiotics or oral/inhaled corticosteroids use for at least 3 months	EC: Nicotine e‐cig daily for at least 6 months with a positive urine cotinine test at baseline	NV: Not using e‐cig for at least 90 days with a negative urine cotinine test at baseline	Urine cotinine test	*Gingival inflammation*: NV: Absent = 63% Present = 36% EC: Absent = 40% Present = 56% *Freq. of brushing teeth*: NV: < 1 = 1% 1 = 21% > 1 = 77/% EC: < 1 = 11% 1 = 39% > 1 = 51%	No eating, no tooth brushing, no flossing, no rinsing and no gum chewing 8 h before sampling	Alpha diversity (observed OTUs, Shannon, Simpson, Inverse Simpson, Fisher, Chao1, ACE, ICE, PD); Beta diversity (Bray–Curtis, Jaccard, Unweighted, Generalised and Weighted UniFrac); taxonomic differential abundance analysis (phylum, genus and species)	NIH grant, and the National Research Foundation of Korea grant funded by the Korea government (MSIT)
Yang et al. (2023), USA	Cross‐sectional	*N* = 36 (NV = 18; EC = 18)	NV: 38.67 ± 7.99/100% F EC: 34.97 ± 7.65/61% F Non‐White were predominant among EC (78%) while White (61%) among NV	Nicotine use: 1–6 mg (69%) 7–12 mg (17%) 13–18 mg (6%) ≥ 25 mg (6%) Don't know (11%) DU (56%): 3.9 ± 3.5 cig/day	NV	Self‐reported	NR	NR	Alpha diversity (Chao1, Shannon); Beta diversity (Bray–Curtis, Jaccard); taxonomic differential abundance analysis (phylum, genus and species)	Hercules Exposome Research Center and the Center for Children's Health, the Environment, the Microbiome, and Metabolomics
Miluna‐Meldere et al. (2024), Latvia	Cross‐sectional	*N* = 18 (NS = 5; CS = 4; EC = 9)	NS: 21–26 (range)/40% F CS: 23–29/0% F EC: 19–23/67% F All northern European *Exclusion criteria*: Systemic diseases, daily alcohol or medication use, pregnancy, antibiotics < 1 year, < 2 years daily use of a single tobacco/nicotine product	E‐cig usage: 1–2 pods/week (*N* = 8), > 2 pods/week (*N* = 1) Nicotine use duration: 5–10 years	NS: Never smoked any tobacco product CS: Tobacco use for 5–10 years; 10–20 cig/day (*N* = 3), > 20 cig/day (*N* = 1)	Self‐reported	*BPE*: NS: 60% (score 1) and 40% (score 2) CS: 100% (score 2) EC: 89% (score 2) and 11% (score 1) No oral mucosal alterations were observed in any group People with active caries, periodontitis (3–4 score) and dentures were excluded (*N* = 14)	No eating, no drinking, no using any tobacco or nicotine product, no brushing teeth for 30 min before sampling	To identify clinical isolates of selected periodontal bacteria: *Porphyromonas gingivalis* , *Tannerella forsythia* , *Prevotella intermedia*, *Fusobacterium nucleatum* , *Fusobacterium periodonticum* , *Porphyromonas endodontalis* , and *Rothia mucilaginosa* .	No external funding
Kurniawan et al. (2025), Indonesia	Cross‐sectional	*N* = 390 (NS = 195; CS = 18; EC = 42; EC‐FS = 37; DU = 98)	NS: 19.7 ± 2.3/77.6% F CS: 20.9 ± 1.9/0% F EC: 20.4 ± 2.3/8.9% F EC‐FS: 20.5 ± 1.5/5.2% F DU: 20.3 ± 2.0/8.3% F *Exclusion criteria*: Systemic diseases and drug and alcohol consumption	EC: E‐cig usage for 1 year minimum	CS: Cig usage for 1 year minimum NS: Had never smoked	Self‐reported	NR	No eating, no drinking, or smoking for 1 h before sampling	To identify clinical isolates of selected bacteria ( *Streptococcus mutans* [16s rRNA], *Porphyromonas gingivalis* [*16s rRNA*]) and fungi ( *Candida albicans* [*18s rRNA*])	The Institute for Research and Community Service of Universitas Trisakti
Liu et al. (2025), China	Cross‐sectional	*N* = 113 (NS = 40; CS = 46; EC = 27)	Only males aged between 18 and 35 years	EC: 3 cartridges/day	NS: Never smoked any tobacco product CS: ≥ 5 cig/day	Self‐reported	People with chronic dry mouth, periodontal pockets ≥ 4 mm, untreated caries/abscesses, precancerous or cancerous oral lesions, candidiasis; halitosis, > 8 missing teeth, gum bleeding > 10% of sites were excluded	No eating, brushing teeth before sampling	Alpha diversity (Chao1, Observed OTUs, Shannon, Simpson, Faith's PD, Pielou's evenness, Good's coverage); beta diversity (Bray–Curtis, Jaccard, Weigheted UniFrac); taxonomic differential abundance analysis (phylum and genus)	Yantai Development Zone Science and Technology Leading Talents Project, the Shandong Taishan Leading Talent Project, the Technology‐based small and medium enterprises innovation capacity enhancement project, the Central leadership of local science and technology development special funds, and the Qilu University of Science, education and industry integration innovation pilot project
Yang et al. (2025), USA	Cross‐sectional	*N* = 70 (NV: 22; EC: 48) Low‐Flow Vapers (*n* = 7) Medium‐flow vapers (*n* = 5) High‐flow vapers (*n* = 5)	NV: 28.5 ± 5.4/59.1% F EC: 29.0 ± 4.5/66.7% F African‐American/Black and Caucasian/white were predominant among EC (43.8%) while African‐American/Black (36.4%) among NV *Exclusion criteria*: Use of NRT or other tobacco products, active marijuana use, pregnancy or lactation, diabetes, HIV infection, recent antibiotic or immunosuppressant use (≤ 60 days), oral prophylaxis within 3 months, or household member already enrolled	EC: Vaping either daily or on at least 20 of the past 30 days; having used ENDS on at least 100 occasions; and having started ENDS use more than 90 days prior to enrollment	NV: Non‐vaping and non‐smoking	Self‐reported	*CPITN score*: NV 0: 4.5% 1–2: 22.7% 3–4: 68.2% EC 1–2: 12.5% 3–4: 87.5%	No eating, no drinking (except for water), for at least 3 h before sampling	Alpha diversity (Chao1 and Shannon); beta diversity (Bray–Curtis); taxonomic differential abundance analysis (phylum and genus)	NIH–NIDCR (National Institute of Dental and Craniofacial Research)

Abbreviations: ACE: Abundance Coverage Estimator; BPE: basic periodontal examination; CEJ: cemento‐enamel junction; CO: carbon monoxide; CPITN: Community Periodontal Index of Treatment Needs; CS: current smokers; CVD: cardiovascular disease; DU: dual users; EC: e‐cigarette users; EC‐FS: e‐cigarette users former smokers; MCS: mild periodontitis current smokers; MEC: mild periodontitis e‐cigarette users; MNS: mild periodontitis nonsmokers; MoCS: moderate periodontitis current smokers; MoEC: moderate periodontitis e‐cigarette users; MoNS: moderate periodontitis nonsmokers; NCI: National Cancer Institute; NIH: National Institutes of Health; NR: not reported; NRT: nicotine replacement therapy; NS: never smokers; NSAIDs: non‐steroidal anti‐inflammatory drugs; NSNP: never smokers with no periodontitis; NSP: never smokers with periodontitis; NV: non‐vapers; PCR: polymerase chain reaction; PD: faith's phylogenetic diversity; SCS: severe periodontitis current smokers; SEC: severe periodontitis e‐cigarette users; SNS: severe periodontitis nonsmokers; ST: smokeless tobacco.

^a^ Xu's study included 119 subjects (40 CS, 40 ES, 39 NS), aged 21 years or older, with a follow up of approximately 6 months.

^b^ Mild periodontitis was defined as ≥ 2 interproximal sites with ≥ 3 mm attachment loss (AL), and ≥ 2 mm interproximal sites with probing depth (PD) ≥ 4 mm (not on the same tooth) or one interproximal site with PD ≥ 5 mm. Moderate periodontitis defined as 2 or more interproximal sites with ≥ 4 mm clinical AL (not on the same tooth), or 2 or more interproximal sites with PD ≥ 5 mm, also not on the same tooth. Severe periodontitis defined as having 2 or more interproximal sites with ≥ 6 mm AL (not on the same tooth), and one or more interproximal site(s) with ≥ 5 mm PD (CDC in collaboration with the American Academy of Periodontology).

### 3.2 General Characteristics

Most included studies were cross‐sectional, with two providing 6‐month follow‐up data (Thomas et al. 2022; Xu et al. 2022). Sample size ranged from 18 to 390 participants, mostly young adults, and the majority were conducted in the United States. Definitions of e‐cigarette use varied, and vaping status was generally self‐reported. Comparator groups included never smokers, current smokers and dual users.

Biological samples comprised saliva, subgingival plaque and mucosal swabs, with 16S rRNA gene amplicon sequencing of different hypervariable regions as the predominant method (*n* = 8); others included meta‐transcriptomics (*n* = 1), shotgun metagenomics (*n* = 1) and targeted qPCR (*n* = 2). Outcomes focused on microbial diversity and composition, including alpha and beta diversity as well as differential abundance across taxonomic levels. Full details about sample collection and processing are reported in Appendix 4.

Most of the studies clearly reported funding from academic or public institutions, except one study, which only stated that no external funding was received (Miluna‐Meldere et al. 2024) and Wang's study who reported funding by research foundation affiliated with the state‐owned China National Tobacco Corporation (Wang et al. 2022). Data transparency was generally high, with publicly available sequencing datasets across eight studies reported in 10 publications (Stewart et al. 2018; Thomas et al. 2022; Pushalkar et al. 2020; Xu et al. 2022; Chopyk et al. 2021; Ganesan et al. 2020; Park et al. 2023; Wang et al. 2022; Ying et al. 2022; Yang et al. 2025).

Further details on the general characteristics of the included studies are provided in Appendix 5.

### 3.3 Main Findings

#### 3.3.1 Alpha Diversity

Findings on alpha diversity were inconsistent across studies and varied by sample type. Most studies reported no significant differences when comparing e‐cigarette users with either never smokers or current smokers. However, a few studies reported significant increases in specific indices among e‐cigarette users compared with never smokers, although this was restricted to the analysis of the salivary microbiota (Pushalkar et al. 2020; Chopyk et al. 2021; Park et al. 2023). Overall, the effect of e‐cigarette use on alpha diversity appeared variable and highly dependent on the sample type (Table 2).

**TABLE 2:** Summary of alpha diversity, beta diversity, and bacterial composition across included studies, grouped by sample type, with comparisons expressed relative to e‐cigarette users (↑ and ↓ indicate higher or lower values in e‐cigarette users compared with the reference group).

Author (year)	Comparator	EC alpha diversity	EC beta diversity	EC differential taxa abundance/bacterial composition	Statistical analysis
Sample type: Saliva					
Stewart et al. (2018)	NS	Not significant for all indices (*p* > 0.05)	Not significant (*p* = 0.382)	Not significant (*p* > 0.05)	Kruskal–Wallis test and Mann–Whitney *U* (alpha diversity and taxonomic relative abundance) PERMANOVA (beta diversity)
	CS	Not significant for all indices (*p* > 0.05)	Not significant (*p* = 0.582)	Not significant (*p* > 0.05)	
Pushalkar et al. (2020) Xu et al. (2022)	NS	Significant only (↑, *p* = 0.023 and *p* = 0.002 for OTUs and PD, respectively) at baseline Significantly ↑ (*p* < 0.05) at baseline in severe periodontitis; not significant at 6‐month follow‐up	Significant (*p* < 0.05) SEC and SNS showed significant differences (V1: *p* = 0.008; V2: *p* = 0.007). No difference for moderate periodontitis (V1: *p* = 0.224; V2: *p* = 0.372)	*Baseline*: ↑ *Actinobacteria* (*p* < 0.01) ↑ *Synergistetes* (*p* < 0.01) ↑ *Veillonella* (*p* = 0.008) Not significant differences for the other taxa *SEC versus SNS*: ↑ *Spirochaetes* (*p* < 0.05) ↓ *Proteobacteria* (*p* < 0.05) Not significant differences for other taxa *MEC versus MNS*: ↑ *Actinobacteria* (*p* < 0.001) ↓ *Bacteroidetes* (*p* < 0.01) Not significant differences for other taxa Periodontal disease‐associated bacterial taxa were enriched in SEC (e.g., *Filifactor*, *p* < 0.005 and *Treponema*, *p* < 0.05) and MEC (*Fusobacterium* spp., *p* < 0.05)	One‐way ANOVA and Tukey's or Kruskal–Wallis test and Mann–Whitney *U*/Dunn's test Wilcoxon signed‐rank test for within‐group (alpha diversity and taxonomic relative abundance) PERMANOVA (beta diversity)
	CS	Not significant (*p* > 0.05)	Significant (*p* < 0.05) SEC/SCS (V1: *p* = 0.359; V2: *p* = 0.316) and MEC/MCS (V1: *p* = 0.539; V2: *p* = 0.545) showed similar composition	*Baseline*: ↑ *Proteobacteria* (*p* < 0.01) ↑ *Fusobacteria* (*p* < 0.024) ↓ *Firmicutes* (*p* < 0.01) ↑ *GN02* (*p* < 0.05) *SES versus SCS*: ↑ *Fusobacteria* (*p* < 0.05) Not significant differences for other taxa (*p* > 0.05) *MES versus MCS*: Not significant (*p* > 0.05) The distribution of periodontal pathogens showed high compositional similarity	
Chopyk et al. (2021)	NS	Significant ↑ (*p* < 0.05) *After 2‐week reduction use*: Not significant (*p* > 0.05)	Jaccard (*p* < 0.05) ↓ Intra‐group differences (*p* < 0.05)	Not significant (*p* > 0.05) *After 2‐week reduction use*: Dominant genera unchanged	Paired or unpaired Wilcoxon tests (alpha diversity and taxonomic relative abundance) ANOSIM tests (beta diversity)
Wang et al. (2022)	NS	Significant ↑ only for Shannon index (*p* < 0.01)	Significant (*p* < 0.05)	↓ *Neisseria* (*p* = 0.027) ↓ *Corynebacterium* (*p* < 0.05) *Porphyromonas*, *Prevotellaceae* (*p* > 0.05)	One‐way ANOVA (alpha diversity and taxonomic relative abundance) PERMANOVA (beta diversity)
	CS	Not significant for all indices (*p* > 0.05)	Significant (*p* < 0.05)	↑ *Prevotellaceae* (*p* < 0.01) ↓ *Neisseria* (*p* = 0.002) *Porphyromonas*, *Corynebacterium* (*p* > 0.05)	
	FS	Significant ↑ only for Shannon index (*p* < 0.05)	Significant (*p* < 0.05)	↓ *Corynebacterium* (*p* < 0.05) *Porphyromonas*, *Corynebacterium*, *Neisseria* (*p* > 0.05)	
Ying et al. (2022)	NS	Not significant for all indices (*p* > 0.05)	Clustered closer to NS than CS (*p* < 0.05)	Not significant (*p* > 0.05)	Kruskal–Wallis (alpha diversity) lmma (taxonomic relative abundance) PERMANOVA (beta diversity)
	CS	Not significant for all indices (*p* > 0.05)		↑ *Herbaspirillum* sp. *Meg3*, *Candidatus Kinetoplastibacterium desouzaii*, *Neisseria sicca* , *Neisseria meningitidis* , and *Neisseria gonorrhoeae* (*p* = 0.034)	
Park et al. (2023)	NV	Significant ↑ for Observed, Fisher, and PD indices (*p* < 0.05)	Not significant (*p* > 0.05)	↓ *Catonella*, *Bergeyella*, *Neisseria*, *Enterococcus* and *Haemophilus* (*p* < 0.001) ↑*Alloscardovia*, *Cryptobacterium* and *Dialister* (*p* < 0.001)	Wilcoxon signed‐rank test (alpha diversity) CLR transformation (taxonomic relative abundance) GLMM‐MiRKAT (beta diversity)
Miluna‐Meldere et al. (2024)	NS	NA	NA	*Samples positive for tested bacteria (N*): *Porphyromonas gingivalis* NS = 0 CS = 0 EC = 1 *Tannerella forsythia* NS = 0 CS = 4 EC = 5 *Prevotella intermedia* NS = 0 CS = 0 EC = 1 *Fusobacterium nucleatum* NS = 1 CS = 4 EC = 8 *Fusobacterium periodonticum* NS = 3 CS = 4 EC = 6 *Porphyromonas endodontalis* NS = 0 CS = 3 EC = 4 *Rothia mucilaginosa* NS = 3 CS = 4 EC = 9	Only descriptive statistics
	CS	NA	NA		
Kurniawan et al. (2025)	NS	NA	NA	*Exclusive use*: ↑ *Porphyromonas gingivalis* (*p* = 0.011) *Streptococcus mutans* , *Candida albicans* Not significant *DU*: *↑ Porphyromonas gingivalis* (*p* = 0.006) *Streptococcus mutans* Not significant *↑ Candida albicans* (*p* = 0.019) *EC‐FS*: *Porphyromonas gingivalis* , *Streptococcus mutans* Not significant *↑ Candida albicans* (*p* = 0.046)	Independent *t*‐tests and one‐way ANOVA testing
	EC‐FS	NA	NA	*Exclusive use*: Not significant for the selected taxa (*p* > 0.05)	
	DU	NA	NA	*Exclusive use*: Not significant for the selected taxa (*p* > 0.05)	
Liu et al. (2025)	NS	Not significant (*p* > 0.05)	Significant (*p* < 0.01)	↓ *Porphyromonas* (*p* < 0.05) ↓ *Streptococcus* (*p* < 0.001) ↓ *Peptostreptococcus* (*p* < 0.001) ↑ *Veillonell*a (*p* < 0.0001) ↑ *Leptotrichia*, *Fusobacterium* (*p* < 0.05)	Kruskal–Wallis test and Dunn's post hoc (alpha diversity) LEfSe (taxonomic relative abundance) PERMANOVA (beta diversity)
	CS	Not significant (*p* > 0.05)	Significant (*p* < 0.01)	↓ *Actinomyces* (*p* < 0.05) ↓ *Peptostreptococcus* (*p* < 0.001) ↓ *Prevotella* (*p* < 0.05) ↑ *Veillonell*a (*p* < 0.0001)	
Sample type: Subgingival plaque (periodontal pockets)					
Ganesan et al. (2020)	NS	↑ (*p* < 0.0001)	Significant (*p* < 0.05)	↑ Gram‐negative facultative (*p* < 0.05) *Abiotrophia*, *Aggregatibacter*, *Eikenella*, *Granulicatella*, *Cardiobacterium*, *Haemophilus*, *Johnsenella*, *Kingella*, *Lachoanaerobaculum*, *Lautropia*, *Leptotrichia*, *Mogibacterium*, *Ottowia*, *Parvimonas*, *Peptostreptococcus*, *Rothia*, *Rhodobacter*, *Selenomonas*, *Veillonella*	Kruskal–Wallis test (alpha diversity) DESeq2 (Wald test) (taxonomic relative abundance) MANOVA (beta diversity)
	CS	↑ (*p* < 0.0001)	Significant (*p* < 0.05)	↑ Gram‐negative facultative anaerobes (*p* < 0.05) ↓ Gram‐negative anaerobes (*p* < 0.05)	
	DU	NR	Clustered closer to DU and FS than NS and CS (*p* = 0.003)	NR	
	FS	NR		NR	
Thomas et al. (2022)	NS	Not significant (*p* > 0.05)	Significant (*p* < 0.05)	*(Baseline + fw)*: ↑ *Fusobacterium* (*p* < 0.05) ↑ *Bacteroidales* [G‐2] (*p* < 0.05) ↑ *Tannarella* (*p* < 0.05) ↑ *Oribacterium* (*p* < 0.05) ↑ *Leptotrichia* (*p* < 0.05) ↑ *Lactobacillus* (*p* < 0.05) ↓ *Streptococcus* (*p* < 0.01) ↓ *Kingella* (*p* < 0.01) ↓ *Scardovia* (*p* < 0.05) ↓ *Ottowia* (*p* < 0.05) ↓ *Aggregatibacter* (*p* < 0.05) ↓ *Granulicatella* (*p* < 0.05) ↓ *Fretibacterium* (*p* < 0.05) ↓ *Neisseria* (*p* < 0.01) ↓ *Porphyromonas* (*p* < 0.01) ↓ *Haemophilus* (*p* < 0.05) ↓ *Gemella* (*p* < 0.001) ↓ *Abiotrophia* (*p* < 0.01) ↓ *Gracilibacteria* (GN02) [G‐1] (*p* < 0.05)	One‐way ANOVA and Tukey's or Kruskal–Wallis test and Mann–Whitney *U*/Dunn's test Wilcoxon signed‐rank test for within‐group (alpha diversity and taxonomic relative abundance) PERMANOVA (beta diversity)
	CS	Not significant (*p* > 0.05)	Significant (*p* < 0.05)	*(Baseline + fw)*: ↑ *Cardiobacterium* (*p* < 0.01) ↑ *Ottowia* (*p* < 0.01) ↑ *Pseudopropionibacterium* (*p* < 0.01) ↑ *Fusobacterium* (*p* < 0.05) ↓ *Streptococcus* (*p* < 0.01) ↓ *Kingella* (*p* < 0.05) ↓ *Scardovia* (*p* < 0.05) ↓ *Veillonella* (*p* < 0.01) ↓ *Megasphaera* (*p* < 0.01) ↓ *Stomatobaculum* (*p* < 0.001)	
Park et al. (2023)	NV	Significant ↑ (*p* < 0.05) for all indices but for Simpson (*p* > 0.05)	Significant (*p* < 0.05) for all indices but for Unweighted UniFrac distance (*p* > 0.05)	↓ *Porphyromonas*, *Capnocytophaga*, *Leptotrichia*, *Actinomyces*, *Corynebacterium*, *Rothia*, *Peptidiphaga*, *Kingella*, *Cardiobacterium*, *Streptococcus*, *Abiotrophia*, *Bergeyella*, *Neisseria*, *Enterococcus* and *Haemophilus* and ↑ *Bacteroidetes_[G‐3]*, *Olsenella*, *Lachnospiraceae_[G‐7]*, *Filifactor*, *Peptostreptococcaceae_[XI][G‐1] Treponema* (*p* < 0.001)	Wilcoxon signed‐rank test (alpha diversity) CLR transformation (taxonomic relative abundance) GLMM‐MiRKAT (beta diversity)
Yang et al. (2025)	NV	Significant ↓ (*p* < 0.05) (Chao index) for high‐puff volume vapers	Not significant (*p* > 0.05)	*EC (total group)*: ↓ *Bacteroidetes* (*p* < 0.05), *Capnocytophaga* (*p* = 0.002), *Corynebacterium* (*p* = 0.006), *Cardiobacterium* (*p* = 0.014), *Bergeyella* (*p* = 0.038), and ↑ *Spirochaetes* (*p* < 0.05), *Treponema* (*p* = 0.044) *Low‐flow vapers*: ↓ *Achromobacter* (*p* = 0.024), *Capnocytophaga* (*p* = 0.039), *Campylobacter* (*p* = 0.044), and ↑ *Spirochaetes* and *Synergistetes* (*p* < 0.05), *Serratia* (*p* = 0.019), *Johnsonella* (*p* = 0.032), *Treponema* (*p* = 0.035) *Medium‐flow vapers* ↑ *Spirochaetes* and *Synergistetes* (*p* < 0.05), *Serratia* (*p* = 0.018), *Fretibacterium* (*p* = 0.019), *Achromobacter* (*p* = 0.034) *High‐flow vapers* ↓ *Granulicatella* (*p* = 0.013) Six taxa (i.e., *Abiotrophia defectiva* , *Leptotrichia*, *Veillonella*, *Stomatobaculum longum*, *Streptococcus*, and *Capnocytophaga*) were positively correlated with puff volume	*t*‐test One‐way ANOVA post hoc Fisher's LSD (alpha diversity) LDM adjusted for age, sex, race, and sugar intake (taxonomic relative abundance) PERMANOVA (beta diversity)
Sample type: Oral swabs (mucosa)					
Stewart et al. (2018)	NS	Not significant for all indices (*p* > 0.05)	Not significant (*p* = 0.886)	Not significant (*p* > 0.05)	Kruskal–Wallis test and Mann–Whitney *U* (alpha diversity and taxonomic relative abundance) PERMANOVA (beta diversity)
	CS	Not significant for all indices (*p* > 0.05)	Significant (*p* = 0.033)	Not significant (*p* > 0.05)	
Chopyk et al. (2021)	NS	Not significant (*p* > 0.05) *After 2‐week reduction use*: Not significant (*p* > 0.05)	Bray–Curtis, Jaccard indices (*p* < 0.05)	↑ *Veillonella* and *Haemophilus* (*p* < 0.05) ↓ *Delftia* (*p* < 0.05) *After 2‐week reduction use*: Dominant genera unchanged	Paired or unpaired Wilcoxon tests (alpha diversity and taxonomic relative abundance) ANOSIM tests (beta diversity)
Yang et al. (2023)	NV	Not significant for all indices (*p* > 0.05)	Significant (*p* < 0.05)	↑ unclassified species of *Veillonella* (*p* = 0.04)	Wilcoxon rank‐sum test (alpha diversity) LDM (taxonomic relative abundance) PERMANOVA (beta diversity)
	DU	Exclusive EC have significant ↓ only for Chao index (*p* = 0.016)	Significant only for Jaccard index (*p* = 0.007)	↓ *Alloprevotella rava* (*p* = 0.014) ↓ *Prevotella maculosa* (*p* = 0.048) ↓ *Tannerella forsythia* (*p* = 0.048)	

Abbreviations: ANOVA: analysis of variance; CLR: centered log‐ratio; CS: current smokers; DU: dual users; EC: e‐cigarette users; EC‐FS: e‐cigarette users former smokers; GLMM‐MiRKAT: Generalised Linear Mixed Model – Microbiome Regression‐Based Kernel Association Test; LDM: Linear Decomposition Model; LEfSe: linear discriminant analysis effect size; lmma: Linear Models for Microarray Data; MCS: mild or moderate periodontitis current smokers; MEC: mild or moderate periodontitis e‐cigarette users; MNS: mild or moderate periodontitis never users; NA: not applicable; NR: not reported; NS: no smokers; NV: no vapers; PERMANOVA: permutational multivariate analysis of variance; SCS: severe periodontitis current smokers; SEC: severe periodontitis e‐cigarette users; SNS: severe periodontitis never users; V1: visit at baseline; V2: visit at 6‐month follow‐up.

#### 3.3.2 Beta Diversity

Across diverse metrics and sample types, multiple studies reported separation between e‐cigarette users and never‐smokers, although effect sizes were not directly comparable. Frequently, e‐cigarette users clustered more closely with current smokers than with never smokers, indicating a partial convergence of microbial profiles between vaping and smoking. However, significant pairwise differences were consistently found among all three groups, underscoring that e‐cigarette users harbour a unique microbiota that is intermediate, sharing features with never smokers and current smokers without fully overlapping with either (Table 2).

#### 3.3.3 Differential Taxa Abundance/Bacterial Composition

At the individual taxa level, some recurrent taxonomic shifts were identified when comparing e‐cigarette users with never smokers, although the results were not consistent and were based on a limited number of studies. Across multiple studies and sample types, e‐cigarette users exhibited higher relative abundance of genera such as *Veillonella*, *Leptotrichia*, and *Fusobacterium* (Thomas et al. 2022; Liu et al. 2025; Pushalkar et al. 2020; Chopyk et al. 2021; Ganesan et al. 2020; Yang et al. 2023; Yang et al. 2025). For several other taxa, findings were inconsistent, with some studies reporting higher abundance, and others reporting no difference and, in some cases, lower abundance. This was particularly evident for *Porphyromonas gingivalis* (Liu et al. 2025; Kurniawan et al. 2025; Park et al. 2023; Wang et al. 2022) and for *Streptococcus*, whose abundance was higher, lower or did not differ, depending on the study and the sample type (Liu et al. 2025; Kurniawan et al. 2025; Park et al. 2023; Yang et al. 2025).

Comparisons between e‐cigarette users and current smokers revealed highly variable patterns of differential microbial taxa. When analysing salivary microbiota, Pushalkar et al. (2020) observed that e‐cigarette users had relatively high levels of *Proteobacteria* and *Fusobacteria* and reduced *Firmicutes* compared with smokers, whereas Liu et al. (2025) reported lower *Actinomyces*, *Prevotella* and *Peptostreptococcus* but higher *Veillonella* in the e‐cigarette group. Wang et al. (2022) found an enrichment of *Prevotellaceae* in e‐cigarette users, whereas *Neisseria* was significantly depleted. Some species such as those classically associated with periodontitis (e.g., *P. gingivalis* and *Fusobacterium nucleatum* ) were enriched in both subgingival plaque of e‐cigarette and cigarette users, suggesting a shared dysbiotic environment (Xu et al. 2022).

Of note, two studies used targeted microbial approaches, specifically qPCR, focusing on selected bacterial taxa rather than the overall microbial community (Miluna‐Meldere et al. 2024; Kurniawan et al. 2025).

In summary, the evidence suggests that e‐cigarette use is associated with alterations in taxonomic abundance of certain bacteria. Some of these differences partially mirror those observed in current smokers, but the patterns remain heterogeneous across studies. This supports the notion of an intermediate microbial state for e‐cigarette users that overlaps with both current and never smokers and appear to be taxon‐ and sample‐specific (Table 2).

### 3.4 Subgroup Analyses

Subgroup analyses examined the influence of vaping pattern, periodontal status and sample type on microbial outcomes. Overall, findings suggested greater differences among individuals with periodontitis and in subgingival plaque samples, while results across vaping patterns and salivary microbiota were less consistent. However, these conclusions are limited by the small number and high heterogeneity of available studies. Detailed results are provided in Appendix 6.

### 3.5 Bias Assessment

**FIGURE 2:**
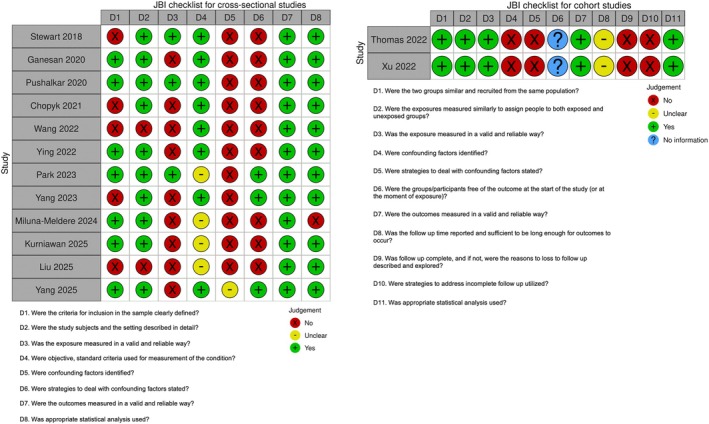
Risk of bias assessment for cross‐sectional and cohort studies using the Joanna Briggs Institute (JBI) checklist.

Risk of bias assessment indicated variable methodological quality across studies, with recurrent issues related to exposure and outcome measurement, confounding control and incomplete reporting (Figure 2). Detailed domain‐level assessments are provided in Appendix 7.

### 3.6 Certainty of the Evidence

The certainty of evidence was rated as very low for all outcomes; detailed GRADE judgements are reported in Table 3 and Appendix 8.

**TABLE 3:** Summary of findings.

Does the oral microbial profile of e‐cigarette users differ from that of never smokers/current smokers?			
Outcome	Effect	Number of participants, studies	Certainty in the evidence
*Population*: Human adults (aged 18 years and older) *Exposure*: E‐cigarette use *Comparison*: Never smoking			
Alpha diversity	Findings were inconsistent across studies and varied by sample type	726 10 non‐randomised (1 cohort; Thomas et al. 2022, 9 cross‐sectional; Stewart et al. 2018; Liu et al. 2025; Chopyk et al. 2021; Ganesan et al. 2020; Park et al. 2023; Wang et al. 2022; Yang et al. 2023; Ying et al. 2022; Yang et al. 2025)	Very low^a^, ^b^, ^c^ ⨁◯◯◯ (due to serious risk of bias, indirectness and inconsistency)
Beta diversity	Across diverse metrics and sample types, multiple studies report separations between e‐cigarette users and never smokers	726 10 non‐randomised (1 cohort; Thomas et al. 2022, 9 cross‐sectional; Stewart et al. 2018; Liu et al. 2025; Chopyk et al. 2021; Ganesan et al. 2020; Park et al. 2023; Wang et al. 2022; Yang et al. 2023; Ying et al. 2022; Yang et al. 2025)	Very low^a^, ^b^, ^c^ ⨁◯◯◯ (due to serious risk of bias, indirectness and inconsistency)
Differential taxa abundance	Some recurrent taxonomic differences were identified (e.g., ↑ *Veillonella*, *Leptotrichia* and *Fusobacterium*), although the results were not consistent and were based on a limited number of studies	726 10 non‐randomised (1 cohort; Thomas et al. 2022, 9 cross‐sectional; Stewart et al. 2018; Liu et al. 2025; Chopyk et al. 2021; Ganesan et al. 2020; Park et al. 2023; Wang et al. 2022; Yang et al. 2023; Ying et al. 2022; Yang et al. 2025)	Very low^a^, ^b^, ^c^ ⨁◯◯◯ (due to serious risk of bias, indirectness and inconsistency)
*Population*: Human adults (aged 18 years and older) *Exposure*: E‐cigarette use *Comparison*: Current smoking			
Alpha diversity	Findings were inconsistent across studies and varied by sample type	446 6 non‐randomised (1 cohort; Thomas et al. 2022, 5 cross‐sectional; Stewart et al. 2018; Liu et al. 2025; Ganesan et al. 2020; Wang et al. 2022; Ying et al. 2022)	Very low^a^, ^b^, ^c^ ⨁◯◯◯ (due to serious risk of bias, indirectness, and inconsistency)
Beta diversity	E‐cigarette users tended to cluster closely with cigarette smokers, suggesting a partial, though not consistently observed, convergence of microbial profiles between vaping and smoking	446 6 non‐randomised (1 cohort; Thomas et al. 2022, 5 cross‐sectional; Stewart et al. 2018; Liu et al. 2025; Ganesan et al. 2020; Wang et al. 2022; Ying et al. 2022)	Very low^a^, ^b^, ^c^ ⨁◯◯◯ (due to serious risk of bias, indirectness, and inconsistency)
Differential taxa abundance	Comparisons between e‐cigarette users and current smokers revealed highly variable patterns of microbial species	446 6 non‐randomised (1 cohort; Thomas et al. 2022, 5 cross‐sectional; Stewart et al. 2018; Liu et al. 2025; Ganesan et al. 2020; Wang et al. 2022; Ying et al. 2022)	Very low^a^, ^b^, ^c^ ⨁◯◯◯ (due to serious risk of bias, indirectness, and inconsistency)

^a^ Serious risk of bias across studies due to limitations in exposure measurement, inadequate identification and control of confounders and, for cohort studies, incomplete follow‐up and insufficient strategies to address missing data and attrition.

^b^ Serious indirectness due to differences in sample types (saliva, oral swabs and subgingival plaque) and in the populations studied (healthy individuals and periodontal patients).

^c^ Serious inconsistency due to the presence of both statistically significant and non‐significant findings across studies.

**FIGURE 3:**
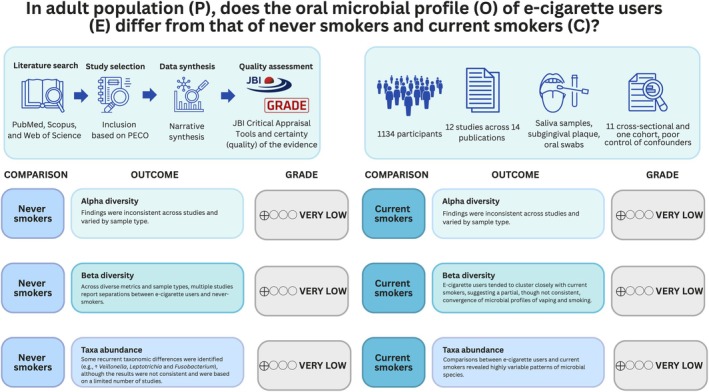
Synthesis of the main findings of the review.

Figure 3 provides an overview of the main findings of the review.

## 4 Discussion

While the effects of combustible tobacco smoking on the oral microbiota are well established (Eggert et al. 2001; Kumar et al. 2011; Wu et al. 2016), those of e‐cigarettes, often positioned as a healthier alternative, remain far more uncertain.

With respect to alpha diversity, the findings were heterogeneous and appeared to vary according to population characteristics and sampling methods. Higher alpha diversity was more frequently observed in studies including periodontal patients (Linden and Mullally 1994; Griffen et al. 2012) and in those analysing salivary or subgingival samples (Pushalkar et al. 2020; Chopyk et al. 2021; Ganesan et al. 2020), whereas studies based on oral swabs often reported no significant differences (Chopyk et al. 2021; Yang et al. 2023).

Regarding beta diversity, most studies reported significant differences in community composition among e‐cigarette users, never smokers and current smokers, suggesting an intermediate microbial profile in vapers, sharing features with both groups.

Regarding differential taxa abundance, some genera (e.g., *Veillonella*, *Leptotrichia* and *Fusobacterium*) were reported as enriched in e‐cigarette users versus never smokers, although evidence was heterogeneous. Comparisons with conventional smokers were mixed, with both shared (Stewart et al. 2018; Wang et al. 2022) and distinct microbial patterns reported (Liu et al. 2025; Ganesan et al. 2020; Wang et al. 2022), limiting firm conclusions.

Given the role of e‐cigarettes as harm‐reduction or cessation tools, comparisons with current smokers are particularly relevant. Some studies suggest that microbial profiles in e‐cigarette users may reflect more favourable oral health conditions than in smokers (Thomas et al. 2022; Liu et al. 2025), with one study reporting greater similarity to never smokers (Ying et al. 2022). However, the evidence remains limited and largely cross‐sectional, highlighting the need for longitudinal studies and for investigations linking microbial profiles to clinically assessed oral health outcomes.

*Veillonella* species are common oral commensals (Rogosa 1964), but their role appears heterogeneous. While some species have been linked to dysbiosis and oral disease (Zhou et al. 2016; Daubert et al. 2018), others may support microbial homeostasis. As noted by Zhou et al. (2021), different *Veillonella* species and strains may either stabilise the oral microbiota or facilitate transitions towards caries and peri‐implantitis. This heterogeneity makes *Veillonella* a relevant target for future studies aimed at identifying species‐specific or functional profiles associated with e‐cigarette use and their clinical implications.

*F. nucleatum* is a key transitional species in the oral microbiome, belonging to the orange complex and implicated in the progression from gingivitis to periodontitis (Socransky et al. 1998). It acts as a microbial bridge between early and late colonisers, facilitating biofilm maturation and inflammatory responses (Huang et al. 2011; Abdulkareem et al. 2023), and has also been associated with extraoral conditions, including ulcerative colitis and colorectal cancer (Su et al. 2020; Piccinno et al. 2025).

Although *P. gingivalis* is strongly associated with periodontitis severity (Chigasaki et al. 2021), studies included in this review reported inconsistent findings regarding its abundance in e‐cigarette users. This variability may reflect a biphasic effect of nicotine exposure, with initial suppression followed by increased growth after prolonged exposure (Shin and Lee 2019; Baek et al. 2012). In addition, the higher levels observed in smokers have been linked to carbon monoxide from cigarette combustion, which is absent in e‐cigarette aerosols (Guglielmetti et al. 2014). These mechanisms may explain the heterogeneity in *P. gingivalis* abundance across vapers with different use patterns and smoking histories.

Overall, variability in the abundance of microbial taxa extends beyond *P. gingivalis* . It is likely influenced by multiple interacting factors, including the status of the e‐cigarette user (i.e., exclusive vs. dual user) (Ganesan et al. 2020), sample types (Park et al. 2023), oral health status (Santacroce et al. 2023), environmental conditions (Li et al. 2014; Murtaza et al. 2019) and individual host variability (Kilian et al. 2016; Cornejo Ulloa et al. 2019). Across studies, the bacterial taxa reported as differing between groups were not consistent, and no reproducible taxonomic pattern associated with e‐cigarette use could be identified.

Vaping behaviour and current or past smoking exposure represent major sources of variability. Species‐specific differences in nicotine affinity and nicotine‐driven modulation of bacterial growth may partly explain the inconsistent findings (Aldakheel et al. 2020). However, nicotine alone may not be the primary driver of microbial changes. Ganesan et al. (2020) reported similar biofilm alterations in response to nicotine‐containing and nicotine‐free aerosols, but not to nicotine alone, suggesting a role for other aerosol components. Consistently, Thomas et al. (2022) found that the duration of e‐cigarette use influenced subgingival microbiota composition more than flavour or nicotine concentration, indicating selection pressure from base components. Another study further showed that reducing e‐cigarette use for 2 weeks decreased alpha diversity without major taxonomic shifts (Chopyk et al. 2021). Together, these findings suggest that aerosol exposure itself may shape the oral microbiota, although isolating specific effects remains challenging given the variability in devices and additives, underscoring the need for standardised products in future studies. Interestingly, Yang et al. (2025) performed stratified analyses according to vaping intensity. Vaping intensity appeared to modulate the subgingival microbiota profile, with high‐flow vapers showing the most pronounced differences compared with non‐vapers, while low‐ and medium‐flow groups exhibited intermediate patterns. However, these findings should be interpreted with caution, as the intensity‐based analyses relied on very small subgroups and were intended for exploratory purposes.

Another relevant factor is the smoking history of e‐cigarette users, which is often self‐reported and incompletely described. As many participants were former smokers, residual microbial alterations may persist, given that oral microbiome recovery after smoking cessation can take 1–2 years (Wu et al. 2016). Consistently, Pushalkar et al. (2020) suggested that nicotine intake levels, rather than vaping per se, may partly account for similarities between exclusive e‐cigarette users and both never smokers and current smokers.

Information on the smoking history of e‐cigarette users was explicitly provided in only four studies (six publications). Moreover, vaping behaviour was self‐reported in over half of the studies, potentially compromising exposure assessment; for example, Stewart et al. (2018) reported undisclosed concurrent cigarette use among participants. Information on e‐cigarette devices was also limited, despite the relevance of device generation and settings, which influence aerosol composition and may affect the oral microbiota (Pushalkar et al. 2020).

To explore heterogeneity, subgroup analyses were conducted by pattern of use (exclusive vs. dual), oral health status and sample type, but results remained inconsistent. Although some studies reported differences between exclusive and dual users, no consistent pattern emerged, possibly due to variability in sampling matrices, which yield distinct microbial profiles (Ganesan et al. 2020; Kurniawan et al. 2025; Yang et al. 2023). In addition, dual use is highly heterogeneous in terms of duration, frequency and relative exposure to e‐cigarettes and cigarettes (Sutton et al. 2022), limiting meaningful aggregated comparisons.

With respect to oral health status, limited evidence suggests that the impact of e‐cigarette use may be more evident in individuals with moderate to severe periodontitis, largely based on a single cohort study (Xu et al. 2022). Other studies included small and heterogeneous samples, limiting comparisons (Miluna‐Meldere et al. 2024; Park et al. 2023). Within these constraints, vaping may interact differently with an already dysbiotic environment, and the more pronounced taxonomic differences observed in subgingival plaque and periodontitis groups likely reflect disease‐related enrichment rather than vaping‐specific effects. These findings underscore the need for closer monitoring of patients with periodontitis and targeted smoking cessation strategies in dental practice (Kowalski et al. 2025; La Rosa, Del Giovane, et al. 2025).

Sample type represents another major source of variability. Saliva showed the most inconsistent results, whereas subgingival plaque appeared more sensitive to vaping‐related differences, particularly for periodontal‐associated taxa, although evidence remains limited. Saliva provides a global snapshot of the oral microbiota but may dilute site‐specific signals due to its heterogeneous composition and variable collection protocols (Yamashita and Takeshita 2017). In contrast, the subgingival microbiome is more stable and less influenced by external factors, potentially explaining its greater consistency in detecting microbial alterations (Huang et al. 2011). As distinct oral habitats host site‐specific communities with known biogeographical differences (Baker et al. 2024), sampling strategy should be carefully considered when interpreting results across studies, and these hypotheses warrant confirmation in larger, well‐controlled cohorts.

Several confounding variables were poorly controlled or unreported, including diet, alcohol and sugar intake, oral hygiene, oral health status (reported in only six studies) and host genetic factors, all known to influence the oral microbiome (Kilian et al. 2016; Li et al. 2014; Murtaza et al. 2019). Only five studies excluded recent antibiotic use (Miluna‐Meldere et al. 2024; Ganesan et al. 2020; Park et al. 2023; Wang et al. 2022; Yang et al. 2025), despite its well‐established impact on microbial composition, underscoring the need for systematic reporting of antibiotic exposure. Biological sex represents another underexplored factor: although sex‐related differences in oral microbiota have been suggested (Haro et al. 2016), findings remain inconsistent (Schenkein et al. 1993; Kim et al. 2020), and future studies should consider sex as a potential moderator of vaping‐related effects.

Beyond biological variability, several methodological factors may contribute to inter‐study differences. Variations in DNA extraction can differentially affect Gram‐positive and Gram‐negative taxa, influencing relative abundance estimates (Fernandez‐Pato et al. 2024). In addition, differences in 16S rRNA variable regions, sequencing platforms, bioinformatics pipelines and reference databases can affect operational taxonomic unit clustering and taxonomic assignment, shaping reported microbial profiles (Sierra et al. 2020). Several methodological steps were incompletely reported, particularly with regard to batch effects, a known source of bias in microbiome research (Ling et al. 2022), for which explicit assessment or control was described in only a few studies (Ganesan et al. 2020; Wang et al. 2022; Yang et al. 2023; Ying et al. 2022). Similarly, negative or extraction blank controls, recommended to identify potential contamination (Hornung et al. 2019), were inconsistently reported. These limitations highlight the need for improved methodological transparency and standardised reporting in future studies.

The certainty of evidence was rated as very low for all outcomes, mainly due to study design, risk of bias and inconsistent findings. Most studies were cross‐sectional, a major limitation for assessing a dynamic ecosystem such as the oral microbiota, as single timepoint analyses do not capture temporal fluctuations related to exposure duration and intensity. These shortcomings reflect those of the included studies, including small samples, incomplete group matching, device heterogeneity and limited control of confounders. Despite these limitations, the findings remain clinically relevant, indicating that e‐cigarette use is associated with a distinct oral microbial profile that shares characteristics with both smokers and non‐smokers, rather than fully resembling either group.

Findings in patients with moderate to severe periodontitis are particularly noteworthy, although based on limited evidence. While no definitive conclusions can be drawn regarding the role of e‐cigarette use, these patients constitute a clinically high‐risk group and warrant careful monitoring and personalised, risk‐oriented dental care strategies (Kowalski et al. 2025). Moreover, as most studies included generally healthy participants, future research should account for systemic conditions such as diabetes, which are known to substantially affect the oral microbiota (Graves et al. 2019).

**FIGURE 4:**
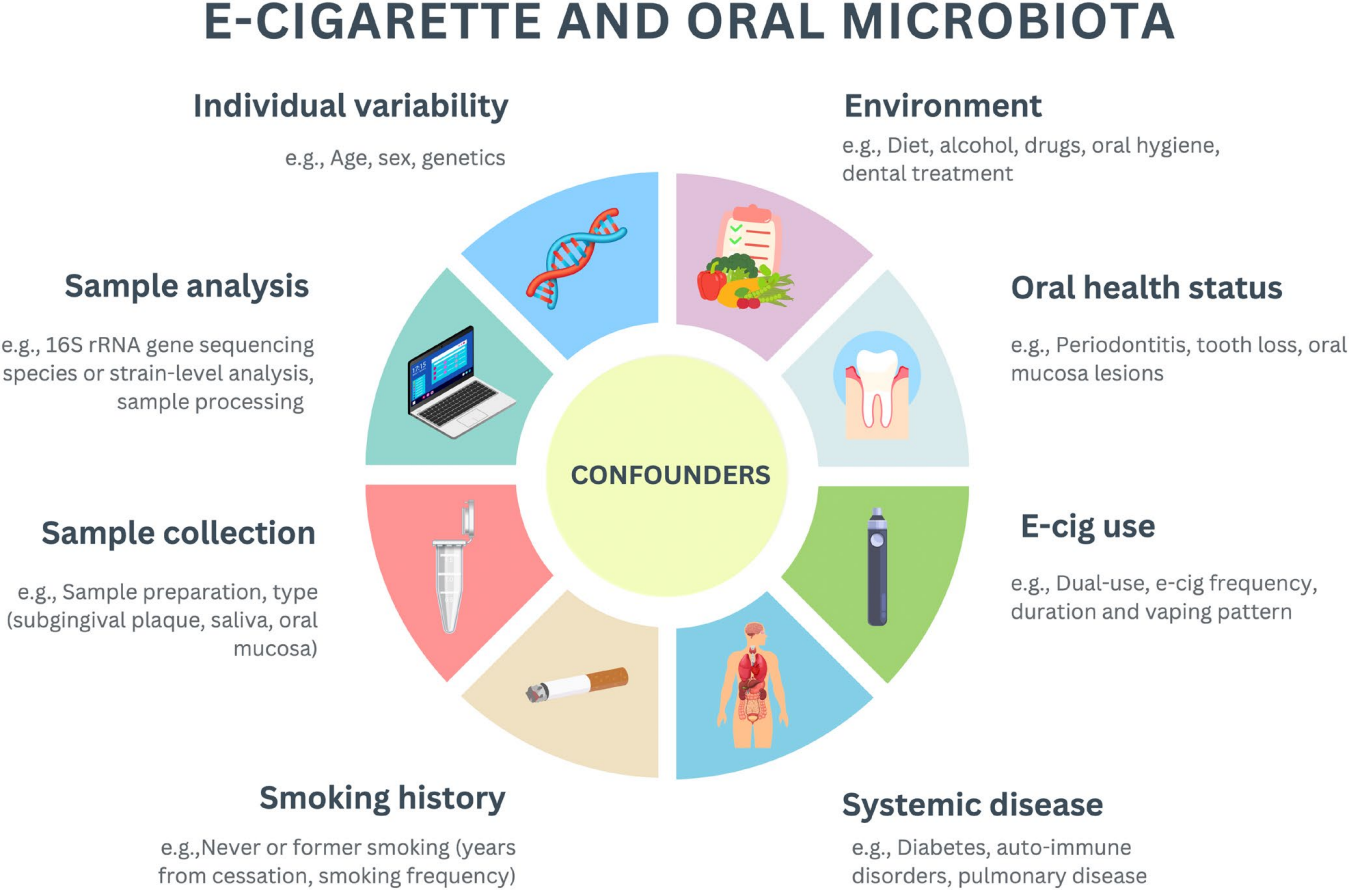
Key confounding factors influencing the relationship between e‐cigarette use and oral microbiota composition, including individual, behavioural, environmental and methodological variables.

From a research perspective, current evidence does not support causal inference, highlighting the need for well‐powered longitudinal studies with standardised exposure definitions and careful control of confounders (Figure 4). Future research should refine sampling strategies, as single matrices may not capture oral ecosystem complexity; parallel sampling of saliva, subgingival plaque and dental or mucosal surfaces is recommended. In addition, reliance on 16S rRNA amplicon sequencing may be insufficient, as higher taxonomic resolution and functional readouts are needed to detect clinically relevant associations. Integrating functional approaches would further clarify the biological relevance of observed compositional differences.

## 5 Conclusions

The available evidence suggests that e‐cigarette use is associated with oral microbiota profiles that partially overlap with, yet remain distinct from, those of never smokers and current smokers, reflecting the specific exposure patterns of vaping. Overall, taxonomic findings should be interpreted cautiously. While some studies report lower enrichment of disease‐associated taxa compared with combustible tobacco, others describe similar microbial profiles, particularly among individuals with periodontitis. Given the influence of oral health status, behavioural and systemic factors, methodological heterogeneity and inconsistent results across studies, the certainty of the evidence is very low and does not support a consistent bacterial signature of e‐cigarette use. Further well‐designed studies with standardised protocols and rigorous control of confounders are needed to clarify the biological relevance of these associations.

## Author Contributions

The study was conceptualised and designed by G.R.M.L.R. and R.P. G.R.M.L.R. was responsible for the methodology, critical analysis and manuscript drafting. L.P.S., E.Z., I.C. and R.P. contributed to data analysis and manuscript preparation. A.S., V.F., P.M.F., J.K. and K.M. contributed to data acquisition and manuscript revision. All authors reviewed and approved the final version of the manuscript.

## Funding

This work was supported by the Department of Clinical and Experimental Medicine, University of Catania (UPB: 6C725202048/2024). The content, selection, and presentation of information in this work, as well as the views expressed, are the sole responsibility of the authors and should not be interpreted as representing the views or positions of the funders.

## Ethics Statement

The authors have nothing to report.

## Consent

The authors have nothing to report.

## Conflicts of Interest

G.R.M.L.R. was awarded a 2026 Scholarship from the Knowledge•Action•Change Tobacco Harm Reduction Programme, an independent public health organisation based in the UK and funded by Global Action to End Smoking, a US‐based independent non‐profit 501(c)(3) grant‐making organisation, for a project focused on oral health. The content of the present paper is unrelated to that project. She was also awarded a Tobacco Harm Reduction Scholarship Programme in 2024/2025. I.C. provides consulting advice to various oral healthcare companies, including Unilever, Philips, Haleon, P&G and Johnson & Johnson. L.P.S. was supported by Chulalongkorn University, second century (C2) high potential professoriate fund at its Faculty of Dentistry. E.Z., A.S., V.F., P.M.F., J.K. and K.M. declare no conflicts of interest. R.P. is a full tenured professor of Internal Medicine at the University of Catania (Italy) and Medical Director of the Institute for Internal Medicine and Clinical Immunology at the same University. He has received grants from U‐BIOPRED and AIR‐PROM, Integral Rheumatology & Immunology Specialists Network (IRIS), Global Action to End Smoking (formerly known as Foundation for Smoke‐Free World), Pfizer, GlaxoSmithKline, CV Therapeutics, NeuroSearch A/S, Sandoz, Merk Sharp & Dohme, Boehringer Ingelheim, Novartis, Arbi Group Srl., Duska Therapeutics, Forest Laboratories, Ministero dell Università e della Ricerca (MUR) Bando PNRR 3277/2021 (CUP E63C22000900006) and 341/2022 (CUP E63C22002080006), funded by NextGenerationEU of the European Union (EU), and the ministerial grant PON REACT‐EU 2021 GREEN‐ Bando 3411/2021 by Ministero dell Universita' e (MUR) – PNRR EU Community. He is founder of the Center for Tobacco Prevention and Treatment (CPCT) at the University of Catania and of the Center of Excellence for the Acceleration of Harm Reduction at the same university. He receives consultancy fees from Pfizer, Boehringer Ingelheim, Duska Therapeutics, Forest Laboratories, CV Therapeutics, Sermo Inc., GRG Health, Clarivate Analytics, Guidepoint Expert Network, and GLG Group. He receives textbooks royalties from Elsevier. He is also involved in a patent application for ECLAT Srl. He is a pro bono scientific advisor for Lega Italiana Anti Fumo (LIAF) and the International Network of Nicotine Consumers Organisations (INNCO); and he is Chair of the European Technical Committee for Standardisation on ‘Requirements and test methods for emissions of electronic cigarettes’ (CEN/TC 437; WG4).

## Data Availability

Data sharing not applicable to this article as no datasets were generated or analysed during the current study.

## Appendix 1 Full Search Strategy for Each Database

**Table:**

Database	Search
Pubmed	(“oral microbiome”[Title/Abstract] OR “oral microbiota”[Title/Abstract]) AND (“e‐cigarette”[Title/Abstract] OR “e cig*”[Title/Abstract] OR “electronic cigarette”[Title/Abstract] OR “Electronic Nicotine Delivery Systems” [MeSH Terms] OR “Electronic Nicotine Delivery Systems” [Title/Abstract] OR ENDS[Title/Abstract] OR vaping[Title/Abstract])
Scopus	TITLE‐ABS‐KEY ((“oral microbiome” OR “oral microbiota”) AND (“e‐cigarette” OR “e cig*” OR “electronic cigarette” OR “Electronic Nicotine Delivery Systems” OR vaping))
Web of Science	ALL = ((“oral microbiome” OR “oral microbiota”) AND (“e‐cigarette” OR “e cig*” OR “electronic cigarette” OR “Electronic Nicotine Delivery Systems” OR vaping))

## Appendix 2 Microbiota‐Specific Criteria for Outcome Measurement Assessment

To address concerns about the domains related to the measure of the outcome (Domain 4 and Domain 7 for cross‐sectional and cohort studies, respectively), microbiota‐specific methodological criteria were evaluated. Specifically, outcome measurement was assessed considering (i) the microbiological outcome definition and measurement approach (e.g., sequencing‐based or qPCR‐based methods), (ii) DNA or RNA extraction kits and library preparation procedures, (iii) bioinformatic quality control pipelines, (iv) reporting of negative or blank controls to detect potential contamination and (v) handling and reporting of batch effects. When two or more of these procedures were not clearly reported or conducted, the corresponding domain was rated as ‘unclear’ or ‘no’.

## Appendix 3 Studies Excluded After Full‐Text Screening

**Table:**

Reference	Reason for exclusion
Adam M, Hasan R. The effects of e‐cigarette use on the oral microbiome. Evid Based Dent. 2023;24:153–4	Study design (commentary)
Coll Y, Geddes A. Is there a significant difference in the oral microbiome in vapers vs. non‐vapers? Evid Based Dent. 2023;24:151–2	Study design (commentary)
Aldakheel FM, Alduraywish SA, Jhugroo P, Jhugroo C, Divakar DD. Quantification of pathogenic bacteria in the subgingival oral biofilm samples collected from cigarette‐smokers, individuals using electronic nicotine delivery systems and non‐smokers with and without periodontitis. Arch Oral Biol. 2020;117:104793	Microbiological method (culture‐based)
Cichonska D, Kusiak A, Piechowicz L, Swietlik D. A pilot investigation into the influence of electronic cigarettes on oral bacteria. Postepy Dermatol Alergol. 2021;38:1092–8	Microbiological method (culture‐based)

## Appendix 4 Sample‐Relating Characteristics Across the Studies

**Table:**

Author (year)	Sample type	Sample collection	Sample analysis	DNA extraction method	Quality control (positive/negative control; batch effect)	Bioinformatics software	Taxonomy database	Data availability
Stewart et al. (2018)	Saliva Buccal mucosa	Saliva, 0.5 mL Buccal swabs	V4 of 16S rRNA gene amplification via PCR and sequencing by Illumina MiSeq platform	PowerMicrobiome RNA Isolation Kit (MoBio, 26000‐50, MoBio Laboratories, Carlsbad, CA, USA)	Negative controls reported (not retained after rarefaction)	UCHIME UPARSE algorithm USEARCH v7.0.1090	SILVA	European Nucleotide Archive (PRJNA413706)
Ganesan et al. (2020)	Subgingival plaque	Subgingival plaque from 15 sites on six maxillary and mandibular anterior teeth by sterile endodontic paper points	Shotgun metagenomic sequencing via Illumina HiSeq platform	Qiagen DNA MiniAmp Kit (Qiagen, Valencia, CA, USA)	Batch effect minimised by random sample allocation across runs and replicate sequencing	Kraken v1.1 MG‐RAST (v3.3.6) PhyloToAST v1.4 QIIME v1.9.0 (University of Colorado, Boulder, CO, USA)	HOMD KEGG	NCBI SRA (PRJNA548383, PRJNA544061, and PRJNA508385)
Pushalkar et al. (2020) Thomas et al. (2022) Xu et al. (2022)	Saliva Subgingival plaque	Stimulated salivaParaffin wax pellet‐chewing stimulated saliva (Gleegum, Verve Inc., Providence, RI) Subgingival plaque collection by sterile Gracey mini‐curette from distal and mesial aspects of eight posterior teeth	V3–V4 of 16S rRNA gene amplification via PCR and sequencing via Illumina MiSeq platform	MoBio PowerFecal DNA Isolation Kit (MoBio Laboratories)	Negative controls were included for all sequencing runs	Cutadapt v1.12 ChimeraSlayer DADA2 FastQC and MultiQC MAFFT QIIME v1.9.0 and QIIME2 (v2020.2) USEARCH	HOMD RefSeq, Version 15.1	NCBI SRA (PRJNA602902, PRJNA771337, PRJNA779977) https://github.com/ Fangxi‐Xu/E‐cigarettes_SGP_Microbiome https://github.com/ Fangxi‐Xu/E‐cigarettes_Saliva_Microbiome
Chopyk et al. (2021)	Saliva Buccal mucosa	Unstimulated saliva (3 mL) collected into a sterile container by drooling for 5 min Omni Swab (Whatman) 6×	V3–V4 of 16S rRNA gene amplification via PCR and sequencing via Illumina MiSeq platform	Qiagen DNeasy Powersoil kit (Qiagen; CA, USA)	Negative extraction controls (sterile PBS) were included	DADA2 QIIME2 (v2019.7)	SILVA	NCBI (PRJNA622970)
Wang et al. (2022)	Saliva	Unstimulated saliva (2–5 mL) spit into a sterile tube; 1 mL obtained by an UltraSal‐2 saliva collection device (Oasis Diagnostics, Vancouver, WA, USA)	V4–V5 of 16S rRNA gene amplification via PCR and sequencing by IonS5TMXL (Ion Torrent, Thermo Fisher)	QIAamp DNA Microbiome Kit (Qiagen, Hilden, Germany)	Samples were sequenced in two batches. Each batch contained three quality control samples, each in triplicate, and a negative control (blank sample for DNA extraction and PCR amplification)	QIIME v1.9.0 RDP Classifier	Greengenes	NCBI (PRJCA002545) Genome Sequence Archive in the BIG Data Center, Chinese Academy of Sciences (CRA002565) http://bigd.big.ac.cn/gs
Ying et al. (2022)	Saliva	Unstimulated saliva collected into a 50 mL tube	RNA‐seq (metatranscriptomics) and sequencing via Illumina HiSeq platform	miRNeasy kit (Qiagen, Germantown, MD)	Library generation batch corrected by using *combat* function in the sva package	Bracken v.1 Cutadapt Kraken v.1 FASTQC TopHat v. 2.1.0	NCBI RefSeq	NCBI GEO (GSE180785)
Park et al. (2023)	Saliva Subgingival plaque	Unstimulated saliva (1 mL) by drooling into saliva Collection Aid (Salimetrics #5016.02) Subgingival plaques were collected by inserting endodontic paper points (Spident #104–0245) into the interproximal gingival sulci of 10 randomly selected teeth until tissue resistance for 10 s	V3‐V4 of 16S rRNA gene amplification via PCR and sequencing by Illumina MiSeq platform	Quick‐DNA Faecal/Soil Microbe Miniprep Kit (D6010)	NR	DADA2 QIIME2 PICRUSt2 USEARCH	eHOMD (16S rRNA RefSeq v. 15.22)	NCBI GEO (GSE201949)
Yang et al. (2023)	Oral mucosa	Swabs from the dorsal tongue, hard palate, buccal mucosa, and keratinized gingiva	V4 of 16S rRNA gene amplification via PCR and sequencing by Illumina MiSeq platform	MoBio PowerMag Soil DNA Isolation Kit	Four template‐free controls Two positive controls, consisting of cloned SUP05 DNA (number of copies = 2 × 10^6^) Batch effect assessed via library size comparison and contaminant filtering	Mothur (v1.39.5)	HOMD (v. 15.1)	Data available from the corresponding author upon reasonable request
Miluna‐Meldere et al. (2024)	Saliva	5 mL saliva collected and aliquoted in Eppendorf tubes.	Targeted PCR of *Porphyromonas gingivalis* , *Tannerella forsythia* , *Prevotella intermedia*, *Fusobacterium nucleatum* , *Fusobacterium periodonticum* , *Porphyromonas endodontalis* , and *Rothia mucilaginosa*	Phenol–chloroform method	NR	NA	NA	The original contributions are included in the article, further inquiries can be directed to the corresponding author
Kurniawan et al. (2025)	Saliva	Unstimulated, accumulated and then spat into a 15 mL Falcon tube over a 10‐min period	Targeted PCR of *Streptococcus mutans* , *Porphyromonas gingivalis* (16S rRNA gene) and *Candida albicans* (18S rRNA gene)	Quick‐DNA Miniprep Plus Kit (Zymo Research, Irvine, CA, USA)	NR	NA	NA	Data available from the corresponding author upon reasonable request
Liu et al. (2025)	Saliva	Unstimulated, allowed to flow into a saliva collector for 5 min	V3–V4 of 16S rRNA gene amplification via PCR and sequencing via Illumina MiSeq platform	OMEGA Soil DNA Kit (M5636‐02) (Omega Bio‐Tek, Norcross, GA, USA)	NR	DADA2 QIIME 2 (v. 2019.4) PICRUSt2	Greengenes, release 13.8 KEGG	Data available from the corresponding author upon reasonable request
Yang et al. (2025)	Subgingival plaque	Subgingival plaque collected using a sterile curette from one randomly selected site per sextant	V3–V4 of 16S rRNA gene amplification via PCR and sequencing via Illumina MiSeq platform	Qiagen DNeasy Powersoil Kit (cat# 12888, Qiagen, Hilden, Germany)	Positive controls (mock microbial community; *Escherichia coli* pellet) and negative controls included during DNA extraction and library preparation	QIIME2 (v2020.2); DADA2; FastQC and MultiQC	HOMD v15.2 (with support from Greengenes v13_8 and SILVA v132)	Bioproject # PRJNA1173730 in https://www.ncbi.nlm.nih.gov/sra

Abbreviations: eHOMD: expanded human oral microbiome database; GEO: gene expression omnibus; HOMD: human oral microbiome database; NA: not applicable; NR: not reported; QIIME2: quantitative insights into microbial ecology 2; SRA: sequence read archive.

## Appendix 5 General Characteristics of the Included Studies

All included studies employed a cross‐sectional design, though two reports (Thomas et al. 2022; Xu et al. 2022) additionally presented 6‐month follow‐up data. Most investigations were conducted in the United States, two in China and one each in Latvia and Indonesia. Sample sizes ranged from 18 to 390 participants, primarily comprising young adults between 20 and 40 years, and female representation varying widely (from 0% to 75%); one study enrolled males exclusively (Liu et al. 2025).

While most cohorts recruited systemically healthy adults, oral health criteria varied: three studies (25%) excluded periodontitis or advanced periodontitis, and one study included patients with at least mild periodontitis, which were reported across three publications (Pushalkar et al. 2020; Thomas et al. 2022; Xu et al. 2022). Nearly half of the studies (*n* = 5) did not specify the periodontal status of the study cohort.

Definitions of e‐cigarette use were inconsistent. Most studies required daily or frequent use of cartridges for ≥ 6 months, though specific thresholds varied from one cartridge (1 mL) to three cartridges per day, with nicotine concentrations ranging from 1 to > 25 mg/mL. Smoking history of e‐cigarette users was explicitly reported in four studies, across six publications (Ganesan et al. 2020; Ying et al. 2022; Pushalkar et al. 2020; Thomas et al. 2022; Xu et al. 2022; Kurniawan et al. 2025). Dual use (i.e., concurrent cigarette and e‐cigarette use) was specifically identified in four studies (Ganesan et al. 2020; Yang et al. 2023; Stewart et al. 2018; Kurniawan et al. 2025). Most studies (*n* = 9) relied solely on self‐report to verify smoking/vaping status, whereas a smaller number also used biochemical confirmation (e.g., exhaled CO, salivary or urinary cotinine).

Comparator groups also varied across the 12 studies. The majority (67%, *n* = 8) analysed both never smokers and current smokers, whereas 42% (*n* = 5) relied solely on never smokers/never vapers. Former smokers were included in 25% (*n* = 3) of the studies and dual users in 17% (*n* = 2).

Substantial methodological heterogeneity was observed. Biological samples included saliva, subgingival plaque and mucosal swabs. Most studies used 16S rRNA gene amplicon sequencing (V3–V4 or V4 region, Illumina MiSeq), one study amplified V4–V5 region of 16S rRNA gene and sequenced using Ion Torrent platform (Thermo Fisher) while others applied shotgun metagenomic sequencing (Illumina HiSeq 4000), RNA‐seq (meta transcriptomics) and targeted selected taxa using PCR (Appendix 4). Outcomes primarily focused on microbial diversity and composition including alpha diversity (i.e., Chao1, ACE, Shannon, Simpson, Faith's PD), beta diversity (i.e., Bray–Curtis, UniFrac, Jaccard), and differential abundance at phylum, genus and species levels.

## Appendix 6 Subgroup Analysis

### Pattern of E‐Cigarette Use

Comparative findings between exclusive e‐cigarette users and dual users (those who use both e‐cigarettes and cigarettes) were mixed. Yang et al. (2023) reported significantly lower Chao1 richness in exclusive users compared with dual users (*p* = 0.016). While in the same study, beta diversity (Jaccard index) showed significant compositional dissimilarity between the groups (i.e., presence/absence of OTUs; *p* = 0.007), though this difference was not observed with the abundance‐weighted Bray–Curtis metric (*p* = 0.31). In line with this, Ganesan et al. (2020) observed that exclusive e‐cigarette users clustered more closely with dual users and former smokers than with never or current smokers (*p* = 0.003).

Taxonomically, Yang reported that exclusive e‐cigarette users were enriched in *Alloprevotella rava* (*p* = 0.014), *Prevotella maculosa* (*p* = 0.048) and *Tannerella forsythia* (*p* = 0.048) compared with dual users (Yang et al. 2023). Conversely, Kurniawan et al. (2025) found that both exclusive and dual users differed from never smokers, with increased detection of *Porphyromonas gingivalis* (*p* = 0.011–0.006) and reduced abundance of *Candida albicans* in dual users (*p* = 0.019), although no significant differences were observed between exclusive and dual use for the taxa assessed.

Overall, inconsistent patterns emerged from comparisons of exclusive e‐cigarette users and dual users. While some studies reported differences in diversity and taxonomic abundance of the selected taxa, no consistent pattern emerged across the cited literature.

### Periodontal Health Status

Only a few studies reported detailed information on participants' periodontal health. One cohort analysed across three reports (Pushalkar et al. 2020; Thomas et al. 2022; Xu et al. 2022) included patients with mild to severe periodontitis. In Xu's stratified analysis (2022), e‐cigarette users with severe disease showed higher alpha diversity compared with never smokers at baseline, although these differences disappeared at the 6‐month follow‐up. Beta‐diversity analyses indicated that among severe cases, e‐cigarette users clustered more closely with smokers, with enrichment of periodontitis‐associated genera such as *Filifactor*, *Treponema* and *Fusobacterium*. Miluna‐Meldere et al. (2024) included participants with early periodontal involvement (Table 1) and detected *Tannerella forsythia* and *Fusobacterium nucleatum* in e‐cigarette and smoker groups but not in controls. Park et al. (2023) reported gingival inflammation in over half of e‐cigarette users, together with higher alpha diversity, distinct beta‐diversity profiles, and widespread taxonomic shifts across saliva and subgingival plaque, when compared to never smokers. Finally, in the study by Yang et al. (2025), Community Periodontal Index of Treatment Needs (CPITN) scores of 3–4, indicative of moderate to severe periodontal conditions, were observed in 87% of e‐cigarette users and 68% of never smokers (*p* > 0.05). Compared with never smokers, e‐cigarette users exhibited alterations in the oral microbiome characterised by a reduction in several commensal taxa and an increased relative abundance of bacteria associated with inflammatory and periodontal disease processes, in the absence of significant differences in alpha or beta diversity.

By contrast, studies that excluded participants with periodontal disease (Ganesan et al. 2020; Liu et al. 2025; Wang et al. 2022) still observed significant beta‐diversity differences for both e‐cigarette users and smokers compared with never smokers. However, findings on alpha diversity and taxonomic abundance in e‐cigarette users were more heterogeneous when compared with both never smokers and current smokers, encompassing both statistically significant and non‐significant results (Table 2). For example, Liu et al. (2025) reported significantly lower *Porphyromon*as among e‐cigarette users compared with never smokers, whereas no significant differences between the same groups were observed in Wang et al. (2022). Overall, although based on a small number of studies, the available evidence suggests that the impact of e‐cigarette use may be more pronounced in individuals with pre‐existing periodontal disease, particularly in moderate to severe cases, where enrichment of classical periodontal taxa and clustering patterns resembling those of cigarette smokers have been observed. In contrast, results in periodontally healthy individuals remain less consistent and heterogeneous, without a clear pattern of microbial alterations.

### Sample Type

When comparing sample types, within the same comparator group, microbial alterations in e‐cigarette users were most consistently observed in subgingival plaque, where beta‐diversity differences were reported across studies and indicated stable group separation. In salivary samples, alpha‐diversity findings were heterogeneous, with some studies reporting significantly higher diversity and others—no difference; beta‐diversity patterns were likewise variable, and taxonomic differences generally limited or inconsistent. In subgingival plaque, alpha‐diversity outcomes were mixed, but taxonomic differences were more pronounced, with enrichment of strict anaerobic Gram‐negative taxa such as *Fusobacterium* and *Tannerella*, together with lower abundance in facultative anaerobic and aerobic Gram‐positives (*Streptococcus*, *Rothia*) and Gram‐negatives (*Kingella*) when compared with non‐smokers. Buccal swabs showed variable beta‐diversity results, with selective enrichments (e.g., *Veillonella*, *Haemophilus*) and reductions (e.g., *Delftia*) compared to never smokers (Chopyk et al. 2021), while alpha diversity was generally unaffected.

Overall, saliva yielded the most inconsistent results, whereas subgingival plaque appeared to be a more sensitive sample for detecting vaping‐related microbial alterations, particularly the enrichment of periodontal disease–associated taxa. However, these findings are derived from a limited number of studies.

## Appendix 7 Bias Assessment

The risk of bias judgement for each study across all domains is presented in Figure 2. Across the cross‐sectional studies (*n* = 12), most showed risk of bias in domains related to exposure measurement (D3) as smoking or vaping status was largely self‐reported rather than objectively verified. Overall, the included studies assessed microbiota‐related outcomes using appropriate and validated methodologies, and outcome measurement was therefore judged as adequate in most cases. However, variability in methodological reporting was observed. In particular, four studies were rated as ‘unclear’ for the outcome measurement domain, primarily due to the absence of explicit reporting on the use of negative or blank controls and the lack of information regarding batch‐effect handling (Park et al. 2023; Miluna‐Meldere et al. 2024; Kurniawan et al. 2025; Liu et al. 2025). Almost all studies were considered at risk for confounding (D5): although two (Park et al. 2023; Yang et al. 2023) reported adjusted analyses accounting for factors such as tooth brushing, age, and gender, none provided a comprehensive strategy to address well‐known confounders, including diet, oral hygiene, characteristics of e‐cigarette devices, pre‐sampling conditions (e.g., eating, smoking abstinence), vaping behaviour, smoking history, and the dual use of e‐cigarettes and conventional cigarettes. Overall, the study by Yang et al. (2025) was judged as having unclear control of confounding. Several key covariates were addressed through statistical adjustment, including age, sex, race and sugar intake, alongside consideration of vaping use patterns and the use of standardised pre‐sampling procedures. However, residual confounding cannot be excluded, particularly with respect to participants' previous smoking history, which was not fully accounted for. Five studies did not clearly state their inclusion criteria, and in two of these (Liu et al. 2025; Wang et al. 2022), demographic characteristics of the samples were insufficiently described. Finally, five studies did not report oral health/periodontal health (Stewart et al. 2018; Chopyk et al. 2021; Ying et al. 2022; Yang et al. 2023; Kurniawan et al. 2025). One study was judged to apply inadequate statistical analysis (D8), relying exclusively on descriptive statistics (Miluna‐Meldere et al. 2024).

The two cohort reports (Thomas et al. 2022; Xu et al. 2022), derived from the same study, were also judged at risk of bias for confounding, as cotinine testing at baseline revealed that approximately 10% of participants in the e‐cigarette group showed biomarkers of cigarette exposure despite being classified as exclusive users. In addition, pre‐sampling preparation (e.g., abstinence from nicotine products or food) was not reported. The duration of follow‐up (6 months) was considered of unclear adequacy to capture meaningful microbiome changes (D8), and reasons for loss to follow‐up were not reported (D9–D10).

## Appendix 8 Certainty of the Evidence

Each outcome was informed almost exclusively by cross‐sectional studies, with the exception of one cohort study. Accordingly, the certainty of evidence started at a low level and was further downgraded to very low due to a serious risk of bias, primarily related to inadequate control of confounding factors, as well as substantial indirectness arising from the limited generalisability of the findings due to differences in study populations (periodontal patients vs. non‐periodontal populations or individuals with unknown oral health status) and sample type.

## References

[jcpe70111-bib-0001] Abdulkareem, A. A. , F. B. Al‐Taweel , A. J. B. Al‐Sharqi , S. S. Gul , A. Sha , and I. L. C. Chapple . 2023. “Current Concepts in the Pathogenesis of Periodontitis: From Symbiosis to Dysbiosis.” Journal of Oral Microbiology 15: 2197779. 37025387 10.1080/20002297.2023.2197779 PMC10071981

[jcpe70111-bib-0002] Adam, M. , and R. Hasan . 2023. “The Effects of e‐Cigarette Use on the Oral Microbiome.” Evidence‐Based Dentistry 24: 153–154. 37875735 10.1038/s41432-023-00946-9

[jcpe70111-bib-0003] Akhi, R. , A. Lavrinienko , M. Hakula , et al. 2025. “Oral Microbiome Diversity Associates With Carotid Intima Media Thickness in Middle‐Aged Male Subjects.” Communications Medicine (London) 5: 66. 10.1038/s43856-025-00773-2 PMC11885836 40050445

[jcpe70111-bib-0004] Aldakheel, F. M. , S. A. Alduraywish , P. Jhugroo , C. Jhugroo , and D. D. Divakar . 2020. “Quantification of Pathogenic Bacteria in the Subgingival Oral Biofilm Samples Collected From Cigarette‐Smokers, Individuals Using Electronic Nicotine Delivery Systems and Non‐Smokers With and Without Periodontitis.” Archives of Oral Biology 117: 104793. 32544646 10.1016/j.archoralbio.2020.104793

[jcpe70111-bib-0005] Baek, O. , W. Zhu , H. C. Kim , and S. W. Lee . 2012. “Effects of Nicotine on the Growth and Protein Expression of *Porphyromonas gingivalis* .” Journal of Microbiology 50: 143–148. 22367949 10.1007/s12275-012-1212-8

[jcpe70111-bib-0006] Baker, J. L. , J. L. Mark Welch , K. M. Kauffman , J. S. McLean , and X. He . 2024. “The Oral Microbiome: Diversity, Biogeography and Human Health.” Nature Reviews. Microbiology 22: 89–104. 37700024 10.1038/s41579-023-00963-6 PMC11084736

[jcpe70111-bib-0007] Benn, A. M. L. , N. C. K. Heng , W. M. Thomson , et al. 2022. “Associations of Sex, Oral Hygiene and Smoking With Oral Species in Distinct Habitats at Age 32 Years.” European Journal of Oral Sciences 130: e12829. 34874583 10.1111/eos.12829

[jcpe70111-bib-0008] Caselli, E. , C. Fabbri , M. D'Accolti , et al. 2020. “Defining the Oral Microbiome by Whole‐Genome Sequencing and Resistome Analysis: The Complexity of the Healthy Picture.” BMC Microbiology 20: 120. 32423437 10.1186/s12866-020-01801-y PMC7236360

[jcpe70111-bib-0009] Chattopadhyay, S. , L. Malayil , J. Chopyk , et al. 2024. “Oral Microbiome Dysbiosis Among Cigarette Smokers and Smokeless Tobacco Users Compared to Non‐Users.” Scientific Reports 14: 10394. 38710815 10.1038/s41598-024-60730-2 PMC11074290

[jcpe70111-bib-0010] Chigasaki, O. , N. Aoyama , Y. Sasaki , et al. 2021. “ *Porphyromonas gingivalis*, the Most Influential Pathogen in Red‐Complex Bacteria: A Cross‐Sectional Study on the Relationship Between Bacterial Count and Clinical Periodontal Status in Japan.” Journal of Periodontology 92: 1719–1729. 33856713 10.1002/JPER.21-0011

[jcpe70111-bib-0011] Chopyk, J. , C. M. Bojanowski , J. Shin , et al. 2021. “Compositional Differences in the Oral Microbiome of E‐Cigarette Users.” Frontiers in Microbiology 12: 599664. 34135868 10.3389/fmicb.2021.599664 PMC8200533

[jcpe70111-bib-0012] Cichonska, D. , A. Kusiak , L. Piechowicz , and D. Swietlik . 2021. “A Pilot Investigation Into the Influence of Electronic Cigarettes on Oral Bacteria.” Postepy Dermatologii I Alergologii 38: 1092–1098. 35126020 10.5114/ada.2020.100335 PMC8802959

[jcpe70111-bib-0013] Coll, Y. , and A. Geddes . 2023. “Is There a Significant Difference in the Oral Microbiome in Vapers vs Non‐Vapers?” Evidence‐Based Dentistry 24: 151–152. 37993687 10.1038/s41432-023-00952-x

[jcpe70111-bib-0014] Cornejo Ulloa, P. , M. H. van der Veen , and B. P. Krom . 2019. “Review: Modulation of the Oral Microbiome by the Host to Promote Ecological Balance.” Odontology 107: 437–448. 30719639 10.1007/s10266-019-00413-x PMC6732124

[jcpe70111-bib-0015] Costa, C. , T. Correia‐de‐Sa , R. Araujo , et al. 2024. “The Oral‐Gut Microbiota Relationship in Healthy Humans: Identifying Shared Bacteria Between Environments and Age Groups.” Frontiers in Microbiology 15: 1475159. 39512939 10.3389/fmicb.2024.1475159 PMC11540997

[jcpe70111-bib-0016] Daubert, D. , A. Pozhitkov , J. McLean , and G. Kotsakis . 2018. “Titanium as a Modifier of the Peri‐Implant Microbiome Structure.” Clinical Implant Dentistry and Related Research 20: 945–953. 30255621 10.1111/cid.12676 PMC6283679

[jcpe70111-bib-0017] Deo, P. N. , and R. Deshmukh . 2019. “Oral Microbiome: Unveiling the Fundamentals.” Journal of Oral and Maxillofacial Pathology: JOMFP 23: 122–128. 10.4103/jomfp.JOMFP_304_18 PMC6503789 31110428

[jcpe70111-bib-0018] Eggert, F. M. , M. H. McLeod , and G. Flowerdew . 2001. “Effects of Smoking and Treatment Status on Periodontal Bacteria: Evidence That Smoking Influences Control of Periodontal Bacteria at the Mucosal Surface of the Gingival Crevice.” Journal of Periodontology 72: 1210–1220. 11577953 10.1902/jop.2000.72.9.1210

[jcpe70111-bib-0019] Fan, X. , K. R. Monson , B. A. Peters , et al. 2024. “Altered Salivary Microbiota Associated With High‐Sugar Beverage Consumption.” Scientific Reports 14: 13386. 38862651 10.1038/s41598-024-64324-w PMC11167035

[jcpe70111-bib-0020] Fernandez‐Pato, A. , T. Sinha , R. Gacesa , et al. 2024. “Choice of DNA Extraction Method Affects Stool Microbiome Recovery and Subsequent Phenotypic Association Analyses.” Scientific Reports 14: 3911. 38366085 10.1038/s41598-024-54353-w PMC10873414

[jcpe70111-bib-0021] Ganesan, S. M. , S. M. Dabdoub , H. N. Nagaraja , et al. 2020. “Adverse Effects of Electronic Cigarettes on the Disease‐Naive Oral Microbiome.” Science Advances 6: eaaz0108. 32518820 10.1126/sciadv.aaz0108 PMC7253170

[jcpe70111-bib-0022] Graves, D. T. , J. D. Correa , and T. A. Silva . 2019. “The Oral Microbiota Is Modified by Systemic Diseases.” Journal of Dental Research 98: 148–156. 30359170 10.1177/0022034518805739 PMC6761737

[jcpe70111-bib-0023] Griffen, A. L. , C. J. Beall , J. H. Campbell , et al. 2012. “Distinct and Complex Bacterial Profiles in Human Periodontitis and Health Revealed by 16S Pyrosequencing.” ISME Journal 6: 1176–1185. 22170420 10.1038/ismej.2011.191 PMC3358035

[jcpe70111-bib-0024] Guglielmetti, M. R. , E. F. Rosa , D. S. Lourencao , et al. 2014. “Detection and Quantification of Periodontal Pathogens in Smokers and Never‐Smokers With Chronic Periodontitis by Real‐Time Polymerase Chain Reaction.” Journal of Periodontology 85: 1450–1457. 24794687 10.1902/jop.2014.140048

[jcpe70111-bib-0025] Haro, C. , O. A. Rangel‐Zuniga , J. F. Alcala‐Diaz , et al. 2016. “Intestinal Microbiota Is Influenced by Gender and Body Mass Index.” PLoS One 11: e0154090. 27228093 10.1371/journal.pone.0154090 PMC4881937

[jcpe70111-bib-0026] Hornung, B. V. H. , R. D. Zwittink , and E. J. Kuijper . 2019. “Issues and Current Standards of Controls in Microbiome Research.” FEMS Microbiology Ecology 95: 1–7. 10.1093/femsec/fiz045 PMC6469980 30997495

[jcpe70111-bib-0027] Huang, R. , M. Li , and R. L. Gregory . 2011. “Bacterial Interactions in Dental Biofilm.” Virulence 2: 435–444. 21778817 10.4161/viru.2.5.16140 PMC3322631

[jcpe70111-bib-0028] Jia, Y. J. , Y. Liao , Y. Q. He , et al. 2021. “Association Between Oral Microbiota and Cigarette Smoking in the Chinese Population.” Frontiers in Cellular and Infection Microbiology 11: 658203. 34123872 10.3389/fcimb.2021.658203 PMC8195269

[jcpe70111-bib-0029] Kilian, M. , I. L. Chapple , M. Hannig , et al. 2016. “The Oral Microbiome – An Update for Oral Healthcare Professionals.” British Dental Journal 221: 657–666. 27857087 10.1038/sj.bdj.2016.865

[jcpe70111-bib-0030] Kim, Y. S. , T. Unno , B. Y. Kim , and M. S. Park . 2020. “Sex Differences in Gut Microbiota.” World Journal of Men's Health 38: 48–60. 10.5534/wjmh.190009 PMC6920072 30929328

[jcpe70111-bib-0031] Kowalski, J. , G. R. M. La Rosa , A. Di Stefano , et al. 2025. “Navigating the Dual Burden of Dental and Periodontal Care in Individuals Who Also Smoke: An Expert Review.” Journal of Dentistry 157: 105744. 40216069 10.1016/j.jdent.2025.105744

[jcpe70111-bib-0032] Kroese, J. M. , B. W. Brandt , M. J. Buijs , et al. 2021. “Differences in the Oral Microbiome in Patients With Early Rheumatoid Arthritis and Individuals at Risk of Rheumatoid Arthritis Compared to Healthy Individuals.” Arthritis and Rheumatology 73: 1986–1993. 33949151 10.1002/art.41780 PMC8596438

[jcpe70111-bib-0033] Kumar, P. S. , C. R. Matthews , V. Joshi , M. de Jager , and M. Aspiras . 2011. “Tobacco Smoking Affects Bacterial Acquisition and Colonization in Oral Biofilms.” Infection and Immunity 79: 4730–4738. 21859855 10.1128/IAI.05371-11 PMC3257914

[jcpe70111-bib-0034] Kurniawan, A. V. , R. Amtha , I. Gunardi , A. Heriandi , and E. F. Sari . 2025. “The Impact of Electronic and Conventional Cigarette Use Towards Saliva Profile and Oral Microbiota in Adolescents.” Asian Pacific Journal of Cancer Prevention: APJCP 26: 309–318. 39874014 10.31557/APJCP.2025.26.1.309 PMC12082429

[jcpe70111-bib-0035] La Rosa, G. R. M. , C. Del Giovane , S. Minozzi , et al. 2025. “Oral Health Effects of Non‐Combustible Nicotine Products: A Systematic Review and Network Meta‐Analysis of Randomized Controlled Trials.” Journal of Dentistry 160: 105910. 40518041 10.1016/j.jdent.2025.105910

[jcpe70111-bib-0036] La Rosa, G. R. M. , A. I. Lorenzo‐Pouso , V. C. A. Caponio , and M. V. Puci . 2025. “Apical Periodontitis in Inflammatory Bowel Disease: A Meta‐Analysis at Patient and Tooth Level.” Frontiers in Dental Medicine 6: 1553914. 40008255 10.3389/fdmed.2025.1553914 PMC11847799

[jcpe70111-bib-0037] La Rosa, G. R. M. , L. P. Samaranayake , E. Zaura , et al. 2025. “Impact of Electronic Cigarette Use on the Oral Microbiome: A Protocol for a Systematic Review of Clinical Studies.” Systematic Reviews 14: 199. 41131626 10.1186/s13643-025-02965-2 PMC12548169

[jcpe70111-bib-0038] Li, J. , D. Quinque , H. P. Horz , et al. 2014. “Comparative Analysis of the Human Saliva Microbiome From Different Climate Zones: Alaska, Germany, and Africa.” BMC Microbiology 14: 316. 25515234 10.1186/s12866-014-0316-1 PMC4272767

[jcpe70111-bib-0039] Linden, G. J. , and B. H. Mullally . 1994. “Cigarette Smoking and Periodontal Destruction in Young Adults.” Journal of Periodontology 65: 718–723. 7608851 10.1902/jop.1994.65.7.718

[jcpe70111-bib-0040] Lindson, N. , A. R. Butler , H. McRobbie , et al. 2025. “Electronic Cigarettes for Smoking Cessation.” Cochrane Database of Systematic Reviews 1: CD010216. 33052602 10.1002/14651858.CD010216.pub4 PMC8094228

[jcpe70111-bib-0041] Ling, W. , J. Lu , N. Zhao , et al. 2022. “Batch Effects Removal for Microbiome Data via Conditional Quantile Regression.” Nature Communications 13: 5418. 10.1038/s41467-022-33071-9 PMC9477887 36109499

[jcpe70111-bib-0042] Liu, J. , Q. Yue , S. Zhang , et al. 2025. “A Pilot Study on Oral Microbiome in Electronic Cigarettes Consumers Versus Traditional Cigarettes Smokers.” Folia Microbiologica (Praha) 70: 147–158. 38954243 10.1007/s12223-024-01185-w

[jcpe70111-bib-0043] Marsh, P. D. 1994. “Microbial Ecology of Dental Plaque and Its Significance in Health and Disease.” Advances in Dental Research 8: 263–271. 7865085 10.1177/08959374940080022001

[jcpe70111-bib-0044] Mason, M. R. , P. M. Preshaw , H. N. Nagaraja , S. M. Dabdoub , A. Rahman , and P. S. Kumar . 2015. “The Subgingival Microbiome of Clinically Healthy Current and Never Smokers.” ISME Journal 9: 268–272. 25012901 10.1038/ismej.2014.114 PMC4274424

[jcpe70111-bib-0045] Miluna‐Meldere, S. , D. Rostoka , R. Broks , et al. 2024. “The Effects of Nicotine Pouches and E‐Cigarettes on Oral Microbes: A Pilot Study.” Microorganisms 12: 12. 10.3390/microorganisms12081514 PMC11356086 39203357

[jcpe70111-bib-0046] Murad, M. H. , R. A. Mustafa , H. J. Schunemann , S. Sultan , and N. Santesso . 2017. “Rating the Certainty in Evidence in the Absence of a Single Estimate of Effect.” Evidence‐Based Medicine 22: 85–87. 28320705 10.1136/ebmed-2017-110668 PMC5502230

[jcpe70111-bib-0047] Murtaza, N. , L. M. Burke , N. Vlahovich , et al. 2019. “Analysis of the Effects of Dietary Pattern on the Oral Microbiome of Elite Endurance Athletes.” Nutrients 11: 1–12. 10.3390/nu11030614 PMC6471070 30871219

[jcpe70111-bib-0048] Nath, S. , P. Zilm , L. Jamieson , P. H. R. Santiago , D. H. K. Ketagoda , and L. Weyrich . 2025. “The Influence of Diet, Saliva, and Dental History on the Oral Microbiome in Healthy, Caries‐Free Australian Adults.” Scientific Reports 15: 18755. 40436959 10.1038/s41598-025-03455-0 PMC12120111

[jcpe70111-bib-0049] O'Leary, R. , G. R. M. La Rosa , and R. Polosa . 2025. “Examining e‐Cigarettes as a Smoking Cessation Treatment: A Critical Umbrella Review Analysis.” Drug and Alcohol Dependence 266: 112520. 39662357 10.1016/j.drugalcdep.2024.112520

[jcpe70111-bib-0050] Page, M. J. , J. E. McKenzie , P. M. Bossuyt , et al. 2021. “The PRISMA 2020 Statement: An Updated Guideline for Reporting Systematic Reviews.” BMJ (Clinical Research Ed.) 372: n71. 10.1136/bmj.n71 PMC8005924 33782057

[jcpe70111-bib-0051] Park, B. , H. Koh , M. Patatanian , et al. 2023. “The Mediating Roles of the Oral Microbiome in Saliva and Subgingival Sites Between e‐Cigarette Smoking and Gingival Inflammation.” BMC Microbiology 23: 35. 36732713 10.1186/s12866-023-02779-z PMC9893987

[jcpe70111-bib-0052] Piccinno, G. , K. N. Thompson , P. Manghi , et al. 2025. “Pooled Analysis of 3,741 Stool Metagenomes From 18 Cohorts for Cross‐Stage and Strain‐Level Reproducible Microbial Biomarkers of Colorectal Cancer.” Nature Medicine 31: 2416–2429. 10.1038/s41591-025-03693-9 PMC12283368 40461820

[jcpe70111-bib-0053] Pushalkar, S. , B. Paul , Q. Li , et al. 2020. “Electronic Cigarette Aerosol Modulates the Oral Microbiome and Increases Risk of Infection.” iScience 23: 100884. 32105635 10.1016/j.isci.2020.100884 PMC7113564

[jcpe70111-bib-0054] Rogosa, M. 1964. “The Genus Veillonella. I. General Cultural, Ecological, and Biochemical Considerations.” Journal of Bacteriology 87: 162–170. 14102850 10.1128/jb.87.1.162-170.1964 PMC276976

[jcpe70111-bib-0055] Santacroce, L. , P. C. Passarelli , D. Azzolino , et al. 2023. “Oral Microbiota in Human Health and Disease: A Perspective.” Experimental Biology and Medicine (Maywood, N.J.) 248: 1288–1301. 10.1177/15353702231187645 PMC10625343 37688509

[jcpe70111-bib-0056] Schenkein, H. A. , J. A. Burmeister , T. E. Koertge , et al. 1993. “The Influence of Race and Gender on Periodontal Microflora.” Journal of Periodontology 64: 292–296. 8387107 10.1902/jop.1993.64.4.292

[jcpe70111-bib-0057] Shigdel, R. , A. Johannessen , H. Lin , et al. 2023. “Oral Bacterial Composition Associated With Lung Function and Lung Inflammation in a Community‐Based Norwegian Population.” Respiratory Research 24: 183. 37438766 10.1186/s12931-023-02491-6 PMC10337198

[jcpe70111-bib-0058] Shin, J. , and S.‐W. Lee . 2019. “The Effect of Cigarette Smoking on *Porphyromonas gingivalis*, a Crucial Periodontal Pathogen.” Oral Biology Research 43: 17–22.

[jcpe70111-bib-0059] Sierra, M. A. , Q. Li , S. Pushalkar , et al. 2020. “The Influences of Bioinformatics Tools and Reference Databases in Analyzing the Human Oral Microbial Community.” Genes (Basel) 11: 11. 10.3390/genes11080878 PMC7465726 32756341

[jcpe70111-bib-0060] Socransky, S. S. , A. D. Haffajee , M. A. Cugini , C. Smith , and R. L. Kent Jr. 1998. “Microbial Complexes in Subgingival Plaque.” Journal of Clinical Periodontology 25: 134–144. 9495612 10.1111/j.1600-051x.1998.tb02419.x

[jcpe70111-bib-0061] Stewart, C. J. , T. A. Auchtung , N. J. Ajami , et al. 2018. “Effects of Tobacco Smoke and Electronic Cigarette Vapor Exposure on the Oral and Gut Microbiota in Humans: A Pilot Study.” PeerJ 6: e4693. 29736335 10.7717/peerj.4693 PMC5933315

[jcpe70111-bib-0062] Su, W. , Y. Chen , P. Cao , et al. 2020. “ *Fusobacterium nucleatum* Promotes the Development of Ulcerative Colitis by Inducing the Autophagic Cell Death of Intestinal Epithelial.” Frontiers in Cellular and Infection Microbiology 10: 594806. 33330137 10.3389/fcimb.2020.594806 PMC7728699

[jcpe70111-bib-0063] Sutton, S. K. , K. O. Brandon , P. T. Harrell , et al. 2022. “Identifying Prospective Subpopulations of Combustible and Electronic Cigarette Dual Users in the United States via Finite Mixture Modeling.” Addiction 117: 2493–2503. 35491736 10.1111/add.15906 PMC9795793

[jcpe70111-bib-0064] Thomas, S. C. , F. Xu , S. Pushalkar , et al. 2022. “Electronic Cigarette Use Promotes a Unique Periodontal Microbiome.” MBio 13: e0007522. 35189698 10.1128/mbio.00075-22 PMC8903898

[jcpe70111-bib-0065] Varghese, J. , and P. Muntode Gharde . 2023. “A Comprehensive Review on the Impacts of Smoking on the Health of an Individual.” Cureus 15: e46532. 37927763 10.7759/cureus.46532 PMC10625450

[jcpe70111-bib-0066] Wang, R. R. , Y. S. Xu , M. M. Ji , et al. 2019. “Association of the Oral Microbiome With the Progression of Impaired Fasting Glucose in a Chinese Elderly Population.” Journal of Oral Microbiology 11: 1605789. 31069021 10.1080/20002297.2019.1605789 PMC6493323

[jcpe70111-bib-0067] Wang, X. , Q. Mi , J. Yang , et al. 2022. “Effect of Electronic Cigarette and Tobacco Smoking on the Human Saliva Microbial Community.” Brazilian Journal of Microbiology 53: 991–1000. 35229279 10.1007/s42770-022-00721-5 PMC9151971

[jcpe70111-bib-0068] Wu, J. , B. A. Peters , C. Dominianni , et al. 2016. “Cigarette Smoking and the Oral Microbiome in a Large Study of American Adults.” ISME Journal 10: 2435–2446. 27015003 10.1038/ismej.2016.37 PMC5030690

[jcpe70111-bib-0069] Xu, F. , S. Pushalkar , Z. Lin , et al. 2022. “Electronic Cigarette Use Enriches Periodontal Pathogens.” Molecular Oral Microbiology 37: 63–76. 34997976 10.1111/omi.12361

[jcpe70111-bib-0070] Yamashita, Y. , and T. Takeshita . 2017. “The Oral Microbiome and Human Health.” Journal of Oral Science 59: 201–206. 28637979 10.2334/josnusd.16-0856

[jcpe70111-bib-0071] Yang, I. , X. He , J. Jeon , et al. 2025. “The Impact of Vaping Behavior on Functional Changes Within the Subgingival Microbiome.” Scientific Reports 15, no. 1: 34374. 10.1038/s41598-025-17121-y. 41038906 PMC12491576

[jcpe70111-bib-0072] Yang, I. , J. Rodriguez , C. Young Wright , and Y. J. Hu . 2023. “Oral Microbiome of Electronic Cigarette Users: A Cross‐Sectional Exploration.” Oral Diseases 29: 1875–1884. 35285123 10.1111/odi.14186 PMC10909585

[jcpe70111-bib-0073] Ying, K. L. , T. M. Brasky , J. L. Freudenheim , et al. 2022. “Saliva and Lung Microbiome Associations With Electronic Cigarette Use and Smoking.” Cancer Prevention Research (Philadelphia, Pa.) 15: 435–446. 35667088 10.1158/1940-6207.CAPR-21-0601 PMC9256774

[jcpe70111-bib-0074] Zhang, Y. , X. Wang , H. Li , C. Ni , Z. Du , and F. Yan . 2018. “Human Oral Microbiota and Its Modulation for Oral Health.” Biomedicine & Pharmacotherapy 99: 883–893. 29710488 10.1016/j.biopha.2018.01.146

[jcpe70111-bib-0075] Zhou, P. , X. Li , and F. Qi . 2016. “Identification and Characterization of a Haem Biosynthesis Locus in Veillonella.” Microbiology (Reading) 162: 1735–1743. 27566661 10.1099/mic.0.000366 PMC5756479

[jcpe70111-bib-0076] Zhou, P. , D. Manoil , G. N. Belibasakis , and G. A. Kotsakis . 2021. “Veillonellae: Beyond Bridging Species in Oral Biofilm Ecology.” Frontiers in Oral Health 2: 774115. 35048073 10.3389/froh.2021.774115 PMC8757872

